# Mesoscopic open-eye core–shell spheroid carved anode/cathode electrodes for fully reversible and dynamic lithium-ion battery models†

**DOI:** 10.1039/d0na00203h

**Published:** 2020-07-09

**Authors:** H. Khalifa, S. A. El-Safty, A. Reda, A. Eid, A. Elmarakbi, M. A. Shenashen

**Affiliations:** National Institute for Materials Science (NIMS) Sengen 1-2-1 Tsukuba Ibaraki 305-0047 Japan sherif.elsafty@nims.go.jp SHENASHEN.Mohameda@nims.go.jp https://www.samurai.nims.go.jp/profiles/sherif_elsafty; Department of Mechanical & Construction Engineering, Faculty of Engineering and Environment, Northumbria University Newcastle upon Tyne NE1 8ST UK

## Abstract

We report on the key influence of mesoscopic super-open-eye core–shell spheroids of TiO_2_- and LiFePO_4_-wrapped nanocarbon carved anode/cathode electrodes with uniform interior accommodation/storage pockets for the creation of fully reversible and dynamic Li-ion power battery (LIB) models. The mesoscopic core–shell anode/cathode electrodes provide potential half- and full-cell LIB-CR2032 configuration designs, and large-scale pouch models. In these variable mesoscopic LIB models, the broad-free-access and large-open-eye like gate-in-transport surfaces featured electrodes are key factors of built-in LIBs with excellent charge/discharge capacity, energy density performances, and outstanding cycling stability. Mesoscopic open-eye spheroid full-LIB-CR2032 configuration models retain 77.8% of the 1^st^ cycle discharge specific capacity of 168.68 mA h g^−1^ after multiple cycling (*i.e.*, 1^st^ to 2000^th^ cycles), efficient coulombic performance of approximately 99.6% at 0.1C, and high specific energy density battery of approximately 165.66 W h kg^−1^ at 0.1C. Furthermore, we have built a dynamic, super-open-mesoeye pouch LIB model using dense packing sets that are technically significant to meet the tradeoff requirements and long-term driving range of electric vehicles (EVs). The full-pouch package LIB models retain a powerful gate-in-transport system for heavy loaded electron/Li^+^ ion storage, diffusion, and truck movement through open-ended out/in and then up/downward eye circular/curvy folds, thereby leading to substantial durability, and remarkable electrochemical performances even after long-life charge/discharge cycling.

## Abstract 2

Mesoscopic super-open-eye core/shell spheroids carved anode/cathode electrodes with interiorly-uniform accommodation/storage pockets for creation of fully-reversible and dynamic Li-ion power battery (LIB)-models.

## Graphical abstract

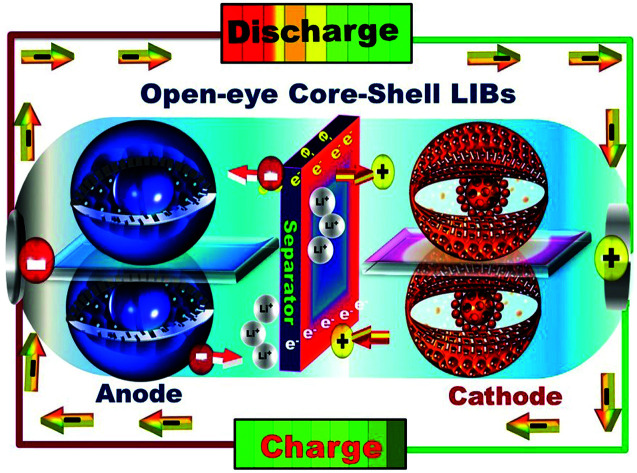

## 1. Introduction

The development of novel energy sources that are economical and environmentally friendly remains a challenge because they must meet the increasing need for green energy in modern society, and assist with overcoming the growing energy crisis in the 21^st^ century.^1–4^ In addition, the scalable, highly precise engineering of rechargeable lithium ion batteries (LIBs) is an extremely advanced technology for producing clean, sustainable, and dense energy for low-power consumption electronics and electric vehicles (EVs). The shortcomings of the current LIBs for EVs are high cost, short-term stability, small driving ranges, and potential hazards in different environments.^5–10^ Accordingly, significant developments in terms of fabrication processes, low material toxicity, material interface surfaces, and exposure sites have been reported to identify the promising control of positive (P) cathode and negative (N) anode materials.^1–5^ These P and N materials can be used to modify electrode surfaces for excellent performance and safety of LIBs.

Among these modified P electrode candidates, olivine-structured LiFePO_4_ (LFPO) is in the forefront and is promising for the fabrication of P electrodes for potential half- and full-scale LIBs.^11,12^ The LFPO structure has two atomic-scale crystal orientations, namely, (i) orthorhombic-rhombohedral (nasicon-type Li_3_Fe_2_(PO_4_)_3_) and (ii) orthorhombic-triphylite (olivine-type) structures.^13,14^ In this context, due to the continuous development of energy storage devices, LFPO's olivine phase is particularly interesting because LFPO P-electrode-mutated half- and full-scale LIBs have revealed that they possess excellent stability, are thermally safe and eco-friendly with low toxicity and low capital cost, and have high specific capacity and charge–discharge cycling stability.^6,15^^.^ The LFPO cathodic materials theoretically display high discharge capacity, with a lithium intercalation voltage plateau at approximately 3.4 V.^16,17^ The LFPO cathode structure also demonstrates low capacity below theoretical levels at room temperature, and irreversible lithiation/delithiation cycles. Furthermore, the surface shortcomings of LFPO enable the impairment of surface conductivities and rate capabilities, poor weak interfacial Li^+^ ion diffusion along the LFPO (LiFePO_4_)/FePO_4_ phases, and low electrode surface tap density.^18–22^ These surface and geometric shortcomings impede their effective implementation for use in emission-free EV LIBs.^22–24^ As a result, intensive efforts have been devoted to overcome the LFPO cathode limitations, including controlled homogeneity of nanoscale particle size,^25,26^ meticulous surface activations and modifications *via* (i) doping of super-valent metal ions,^26–29^ (ii) robust coating with highly conductive carbon nanoparticles,^19,30–37^ and (iii) insertion of conductive additives.^27,38^

Currently, the growing demands for developing LFPO nanoelectrodes have led to promising thresholds for building cathode electrodes with multiplex open-gate surfaces, leading to greater Li^+^ ion diffusivity, and subsequently improved LIB electrochemical performances.^27,39^ Although the nanoscale LFPO morphology sizes have been considered as key influences of the kinetic limitations of electrons/Li^+^ ions, the small size of the LFPO-cathode morphology resulted in the creation of potential half- and full-scale LIBs with low volumetric energy density. Along with this LFPO cathode development, sustainable cathode electrode-integrated LIB systems with hierarchical nanoarchitectures, multi-exposed surface facets, axial dimensions/directions, and surface interfaces and ridges may provide promising storage solutions for LIBs with high gravimetric energy density and specific charging-discharging capacity.^40,41^

To achieve long-range driving of an EV with a LIB with excellent power, retention of the dense and reactive exposure surfaces of cathode- and anode-integrated LIB models is crucial. To achieve low-cost anode electrode fabrication, some alternative metal oxide materials were used to determine gap functionalities using low-capital process cost, active oxide materials, and the development of promising anode electrodes for potential LIBs.^42^ Among these oxides, titanium(iv) dioxide TiO_2_ (TO)-anatase structures are significant in the manufacture of negative (N) anode electrode materials. Due to its naturally occurring advantages including high safety, economics, eco-friendly, and low surface energy polarization, the TiO_2_ (TO)-anatase anode exhibited unique electrochemical performances with high stability of its life cycling.^43–47^ The high cycling stability and reversibility of the anode N electrode design can be attributed to the facile structural phase transition from TO-tetragonal *I*4_1_/*amd* symmetry to lithium-rich phase symmetry with *Imma*-orthorhombic Li_0.5_TiO_2_ structures.^48^ Thus, if the fabricated TO-tetragonal *I*4_1_/*amd* materials can be hybridized with hierarchical building blocks, multifunctional interface surfaces, a variety of binding site interactions, and mobile phase surface topographies, then the TO-anodic electrodes would offer highly optimized LIB designs for a high specific energy density battery and outstanding rate capability of rechargeable batteries.

We report a tailored fabrication of diverse ranges of super-open-mesoeye anode/cathode electrode tectonics and their modulation in half- and full-scale LIB-CR2032 designs, and large-scale dynamic pouch-type models. These half-, full-, and large-scale LIB super-open mesoeye models have been tailored along anatase TiO_2_@nanocarbon shells (ETO@nano-C) as anodic N-electrodes. Moreover, a variety of 3D LiFePO_4_-wrapped nano-C layer (3D-LFPO@nano-C) projections can be used as cathode P electrodes. The mesoscopic open-eye hollow spheroid cathodes including well-designed spheroidal spiky-ball-wrapped 3D cuboid mosaics (SSB@nano-C), mesoporous nanoscale spheres (MS@nano-C), and doubly conjugated nanospheres with multi-open holes (DCS@nano-C) lead to the creation of variable LIB geometrics and models. Remarkably, the SSB@nano-C provided a favorable half-cell cathodic candidate architecture due to the most optimal conductivity, and excellent discharge reversible through discharge (lithiation)/charge (delithiation) processes along the electrolyte/electrode interfaces. The integrated SSB@nano-C cathode//ETO@nano-C anode is a full-scale LIB that demonstrates outstanding features associated with capacity retention, stability, cycling, and coulombic effectiveness until 2000 cycles over voltage range 0.8–3.5 V *vs.* Li/Li^+^. The markedly designated full-scale SSB@nano-C//ETO@nano-C LIB sets achieved a 77.8% capacity retention of its 1^st^ cycle discharge specific capacity of 168.69 mA h g^−1^, and efficient coulombic performance of approximately 99.6% after multiple cycling (*i.e.*, 1^st^ to 2000^th^ cycles) at 0.1C. The full-scale SSB@nano-C//ETO@nano-C CR2032-coin LIB models exhibit a high specific energy density of approximately 165.66 W h kg^−1^ at 1.0C, thereby leading to the long driving range required by EVs. Moreover, a dynamic pouch LIB model can be controlled by densely ordered collar packing of CR2032-coin cell sets that are vertically connected and stacked in layers. In this pouch-type module, the rational control of the SSB@nano-C/ETO@nano-C electrode mass balancing capacity (P/N)_Cap_ ratio loads inside each packed collar coin-cell is crucial for LIB tradeoff models. Our full-pouch package LIB models offer superb areal discharge capacity and volumetric energy densities, and affordable free-space storage stability for force-driving LIB-EV applications.

## 2. Experimental section

### 2.1. Synthesis of 3D-LFPO (core)@nano-C (shell) cathode and ETO (core)@nano-C (shell) anode materials

#### 2.1.1. Fabrication of ETO-nanocore sphere materials

The anode core materials with mesoporous anatase open-eye TiO_2_ (ETO-nanomaterials) were fabricated by controlling the growth rate mechanism. Thus, 10 ml ethylene glycol (0.5 ml min^−1^) was added dropwise to a homogenous solution of titanium(iv) isopropoxide : water : ethanol : acetonitrile mixture with a volumetric amount ratio (ml) of 1 : 1.5 : 2.5 : 2.0, respectively. To this component, 0.1 ml ammonia solution was added dropwise at 0.01 ml min^−1^ under vigorous stirring for 3 h. The thermodynamic growth of TO seeds can be controlled to form mesoscopically shaped ETO spheroids using a hydrothermal treatment pattern. In this designed pattern, a time-dependent thermal treatment (*i.e.*, at 170 °C for 12 h) of the as-made components was carried out to assist the final formation of ETO structures. The ETO materials were collected and washed with a water–ethanol solution. The dried anatase open-eye TiO_2_ materials were formed under a designated thermal treatment pattern at 600 °C for 3 h.

#### 2.1.2. Fabrication of 3D-LFPO-nanocore sphere materials

Various 3D-LFPO-nanocore materials are fabricated by controlling the iron(iii) precursors in composition synthesis domains. For instance, to design SSB-positive electrode materials and as an example of 3D-LFPO-nanocore materials, the SSB synthesis component is controlled with composition ratios of 3 : 1 : 1 for Li : Fe : P elements (*i.e.*, Li_2_SO_4_/lithium sulfate : FeCl_3_/iron(iii) chloride : H_3_PO_4_/phosphoric acid) at pH 7. In typical synthesis, the dropwise addition of H_3_PO_4_ solution (5 ml H_2_O/2.55 ml C_2_H_5_OH) is carried out at a rate of 0.5 ml min^−1^ in FeCl_3_ solution (5 ml H_2_O/2.55 ml C_2_H_5_OH/3 ml C_2_H_6_O_2_, ethylene glycol) under stirring for 1 h. Then, the Li_2_SO_4_ solution (in 10 ml H_2_O/5 ml C_2_H_5_OH/6.0 ml C_2_H_6_O_2_ formed at 30 °C) is added dropwise to the FeCl_3_/H_3_PO_4_ mixture. The thermodynamic growth of 3D-LFPO@nanoseeds can be controlled to form mesoscopically shaped SSB spheroids by the use of a hydrothermal treatment pattern. In this designated pattern, a time-dependent thermal treatment (*i.e.*, at 170 °C for 12 h) of the as-made components is carried out to assist the final formation of SSB structures. The SSB solid products were collected and washed with a H_2_O/C_2_H_5_OH solution. The dried open-entrance-mouth SSB powder product was formed under a thermal treatment pattern at 600 °C for 3 h. The variable 3D-LFPO@nano materials with MS and DCS morphologies are typically fabricated under similar procedures of SSB structure fabrication using different iron(iii) precursor sources, Fe_2_(SO_4_)_3_/iron(iii) sulfate hydrate, and Fe (NO_3_)_3_/iron(iii) nitrate nonahydrate, respectively.

#### 2.1.3. Fabrication of inorganic-core/carbon-shell sphere hybrid cathode and anode materials

To design 3D-LFPO (core)@nano-C (shell) cathode and ETO (core)@nano-C (shell) anode materials, nano-carbon surface wrapping of core 3D-LFPO or ETO open-eye spheroids occurred *via* a post-decoration process, in which the outer sphere cores are depressed by carbon shells. In a typical controlled procedure of 3D-LFPO (core)@nano-C (shell) cathode and ETO (core)@nano-C (shell) anode production, the fabricated SSB, MS, DCS, and ETO core-spheroid powders are sonicated in glucose solution (5 w/w%) for 15 minutes. Microwave irradiation of inorganic core/nano-carbon-shell materials is carried out at 80 °C for 0.5 h with continuous stirring. The collected core/nano-carbon-shell samples are washed and then dried for 12 h at 60 °C. Robust inorganic LFPO or ETO core/nano-carbon-shell materials can be achieved by treatment with a thermal pattern. In this thermal pattern, the core/shell anode and cathode samples are calcined at 350 °C for 0.5 h under an Ar atmosphere, and then continuously calcined for 2 h at 600 °C with heat ramping of 5 °C min^−1^. The mesoscopic super-open-eye core/shell spheroids are considered as anodic ETO@nano-C and cathodic SSB@nano-C, MS@nano-C, and DCS@nano-C materials. In the anode//cathode electrode design, the decoration of the ETO@nano-C anode and the SSB@nano-C, MS@nano-C, and DCS@nano-C cathode electrodes would improve the heterogeneous surface composites, interfaces, and dynamic mobility sites, thereby leading to facile electron and Li^+^ ion transport, and the formation of electronic/conductive layers (Fig. S1–S3†).

### 2.2. Fabrication of mesoscopic super-open-eye core/shell spheroid cathode//anode electrodes

The mesoscopic super-open-eye core/shell spheroids of the integrated 3D-LFPO@nano-C (such as SSB@nano-C, MS@nano-C and DCS@nano-C) cathode and ETO@nano-C anode materials were incorporated into 10 μm-thick aluminum (Al) and 8 μm-thick copper (Cu) foil for fabrication of working positive (P) and negative (N) electrodes, respectively (see Fig. S1†). To practically engineer the P and N working electrodes using an active 3D-LFPO@nano-C cathode and ETO@nano-C-anode materials, a mixture of the active cathode or anode materials/carbon-(C) black/polyvinylidene fluoride (PVDF) was combined with an equivalent mass fraction ratio of 0.75 : 0.15 : 0.1, respectively. A slurry from the mixture was formed by *N*-methyl-2-pyrrolidone (NMP)-assisted solvent under stirring for 1 h. The casting of cathode and anode slurry composites into 10 μm-thick aluminum (Al) and 8 μm-thick copper (Cu) film-foils enabled fabrication of the P (cathode) and N (anode) electrodes, respectively. The as-made super-open-eye anode/cathode disc-like films were then dried for 12 h at 60 °C. The loaded mass amount of the active site cathode and anode materials along the Al and Cu film area was 14.87 and 6.99 mg cm^−2^, respectively, leading to remarkable electrochemical performance of LIB anode/cathode electrode designs in both half- and full-cell super-open-mesoeye models.

### 2.3. Configuration of LIB half- and full-cell super-open-mesoeye models

We fabricated LIB half- and full-cell super-open-mesoeye models designated in CR2032 coin cells (see Fig. S1†) using super-open-mesoeye 3D-LFPO@nano-C (such as SSB@nano-C, MS@nano-C, and DCS@nano-C) cathode P electrodes, and ETO@nano-C anode N electrodes. The unique configuration of super-open-eye core/shell anode and cathode electrodes into both half and full LIB models under specific experimental sets and protocols enabled high electrochemical performances (see ESI S1†). The super-open-eye LIB CR2032 coin cells were formulated with the following components: 16 mm circular-shaped Li-foil electrodes (*i.e.*, counter and reference electrodes), 16 mm circular Al and Cu foils as P and N working electrodes, and a 20 mm circular microporous polymeric membrane separator. We also added 1 M conductive electrolyte solution of lithium hexafluorophosphate (LiPF_6_) dissolved in a (CH_2_O)_2_CO, ethylene carbonate/C_5_H_10_O_3_, diethyl carbonate mixture with 1 : 1 v/v ratio. Prior to the formulation of the CR2032 coin-cell electrode components, we enhanced the mechanical and electrical functionalities of both the dried Li foil reference and super-open-mesoeye Al- and Cu-film electrodes. Thus, the pressing of the dried film electrodes was carried out between twin rollers using the CR20XX crimper series.

Circular perforated electrodes (*i.e.*, 16 mm diameter for Li-chip reference or counter electrodes, and super-open-mesoeye anode/cathode working electrodes) and 20 mm porous-membrane separators were prepared and combined into half and full LIB super-open-mesoeye CR2032-type coin models. We used a crimping machine for coin-cell pressing. The crimper of the CR20XX coin-cell series was applied under precise mechanical treatment conditions such as a clean glove box and under a continuous flow of Ar gas. This packing process provided dense and multi-scale circular 2032 coin-cell electrodes for the enhancement of electrical contact between the super-open-eye solid-electrode surfaces and electrolyte interfaces (SEI) (see ESI S1–S10†). Our protocol for a CR2032-type coin cell design offers a remarkable battery with high mechanical strength, and solid electrical contact between the P and N electrode surfaces and current collector features, and active packing density electrode surfaces for multiple charge–discharge cycles.

### 2.4. Dynamic, super-open-mesoeye pouch LIB models

To fabricate the super-open-eye pouch LIB models, the configured sets can be built up within multiple rolls of coin cells, and then packed into a stacking collar, and these were named as pouch LIB models (see ESI S2 and S3†). In the pouch LIB models, the multi-scale fabrication in terms of dimensions and mass amounts of ETO@nano-C anode/3DLFPO@nano-C cathode electrodes is controlled to obtain tradeoff values for a battery with greater safety and high specific energy density that are primarily required to power EVs with an extended driving range. The stacking sequence of super-open-mesoeye N and P electrodes can be designated in well-packed and dense layers of ETO@nano-C anodes (5 layers/10 sides)//SSB@nano-C cathodes (6 layers/10 sides) oriented in coin cells. The loaded mass ratio (g/g) of both super-open-mesoeye N anode and P cathode electrodes inside the pouch LIB configurations is 0.75 : 1.0, respectively. Furthermore, the full-scale dimensions of N and P electrodes are designated as 35 mm (width), 55 mm (length), and 2.5–3 mm (thickness) in such a pouch LIB model. Thus, the total area of the super-open-mesoeye N anode and P cathode electrodes imprinting the pouch LIB cells are 143 and 150 cm^2^, leading to mass amount stacking of 6.99 and 14.87 mg cm^−2^, respectively. The pouch LIB design indicates that the areal discharge capacity is 1.24 and 1.25 A h cm^−2^ for both super-open-mesoeye ETO@nano-C anode/3DLFPO@nano-C cathode electrodes, respectively (see ESI S2 and S3†).

## 3. Results and discussion

### 3.1. Super-open-mesoeye LFPO@nano-C and ETO@nano-C material carved electrodes and cells

The key factors of mesoscopically shaped open-eye spheroids influence the potential fabrication of variable mesoscopic LIB models, broad free-access surfaces, multi-diffusive and large open-eye like a gate-in-transport diffusion, and electrochemical performances. Various cathodic 3D-LFPO-nanocore materials including SSB, MS, and DCS have been fabricated based on varying the iron compound, such as FeCl_3_/iron(iii) chloride, Fe_2_(SO_4_)_3_/iron(iii) sulfate hydrate, and Fe (NO_3_)_3_/iron(iii) nitrate nonahydrate, in the composition synthesis domains (Li_2_SO_4_/H_3_PO_4_ mixture), respectively. The thermodynamic growth of anodic TO-seed materials with an anatase open-eye ETO nanocore can be controlled under a hydrothermal treatment pattern.

To explore the super-open-eye electric sensibilities, conductive electrode narratives, and structurally stable modes of the anode/cathode electrodes, a nano-carbon surface wrapping protocol for core 3D-LFPO or ETO open-eye spheroids was implemented *via* a post-decoration process. The exterior sphere coating and dressing consisting of 3–5 nm carbon shell layers enabled fabrication of the 3D-LFPO (core)@nano-C (shell) cathode and ETO (core)@nano-C (shell) anode materials (ESI S3 and S11†). Our fabrication route provides evidence that structurally stable ETO@nano-C anode/3D-LFPO@nano-C cathode electrodes can be controlled with mesoscopically shaped open-eye spheroids, heterogeneous surface roughness, and interior uniform accommodation/storage space pockets (*i.e.*, surface mesogrooves and mesoeye entrances, and interior innumerable caves and core hollow nests).

**Scheme 1:**
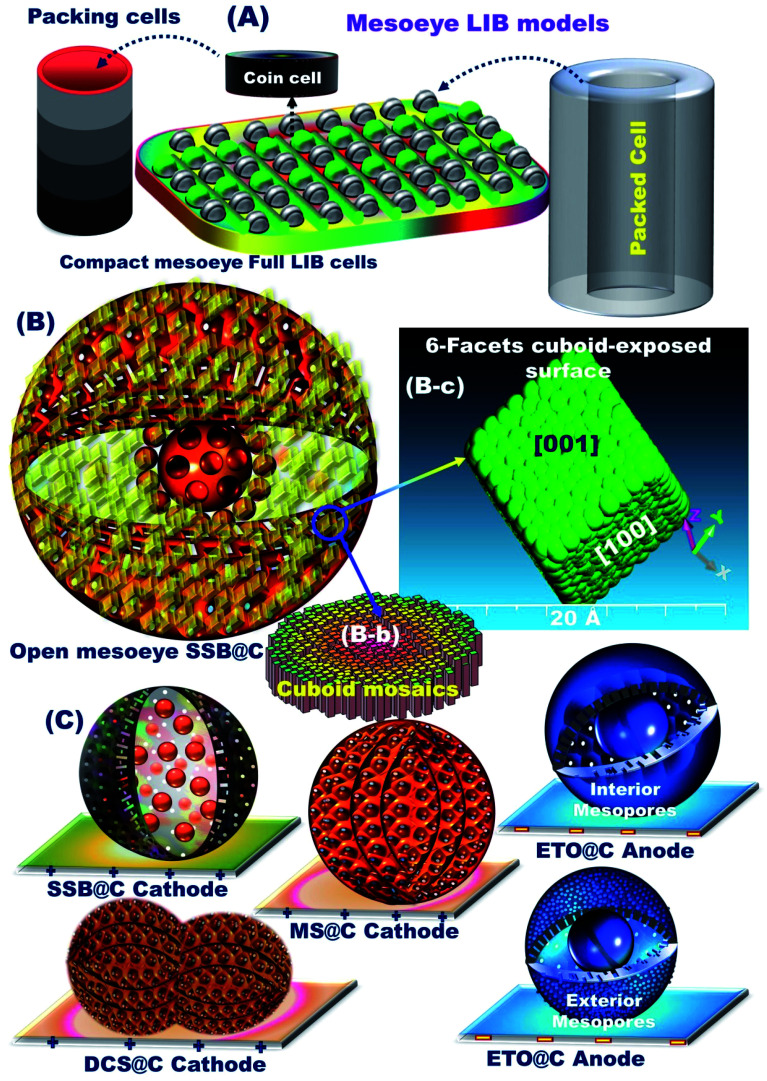
(A) Well fabricated mesoeye LIB based on full-packed coins for manufacturing of rechargeable pouch LIB models for EV applications. (B) The structural control super-open-mesoeye SSB@nano-C-cathode with cuboid-tile geometry (B-b) for electrochemical effectiveness and fully reversible capability for LIBs, and (B-c) a representative model of the parametric {001}-crystal plane with 6-facets of cuboid tile geometry. (C) The full cell configuration of super-open-eye spheroid (cathode/anode) electrodes such as SSB@nano-C, MS@nano-C, and DCS@nano-C cathodes, and the ETO@nano-C anode.

The full-package configuration battery models for super-open-mesoeye half- and full-type LIB-coin-cells and stacking pouch-LIB designs can be generated with (i) a powerful and large open-eye like a gate-in-transport system for heavy electron/Li^+^ ion storage, diffusion, and accommodation, (ii) super-open-eye egress/ingress, out/in, and then up/down, circular/curvy movement folds for fully reversible, dynamic LIB models, (iii) substantial withstanding surface topographies against life charge/discharge cycles, and (iv) real and tangible non-resistance spreading electrons/Li^+^ ion for potential diffusions and occupant loads (Scheme 1).

### 3.2. Fabrication of super-open-mesoeye LFPO@nano-C and ETO@nano-C composite geometrics

**Fig. 1:**
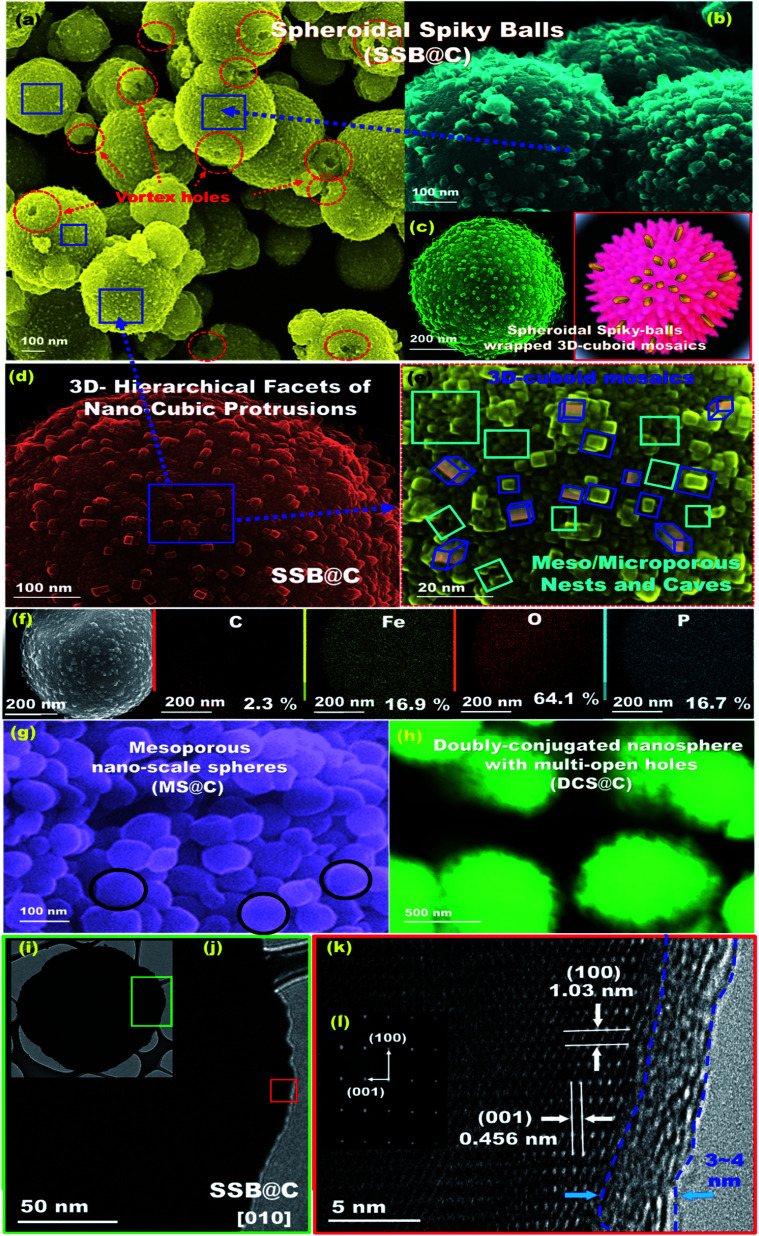
Microscopic analysis patterns based on FE-SEM (a–e and g and h), EDX (f), HR-TEM (i–k), and ED (l) images of mesoscopic super-open-eye core/shell spheroid SSB@nano-C, MS@nano-C, and DCS@nano-C cathodes. (l) The selected area of the ED pattern image recorded along the [010] plane of SSB@nano-C geometrics.

**Fig. 2:**
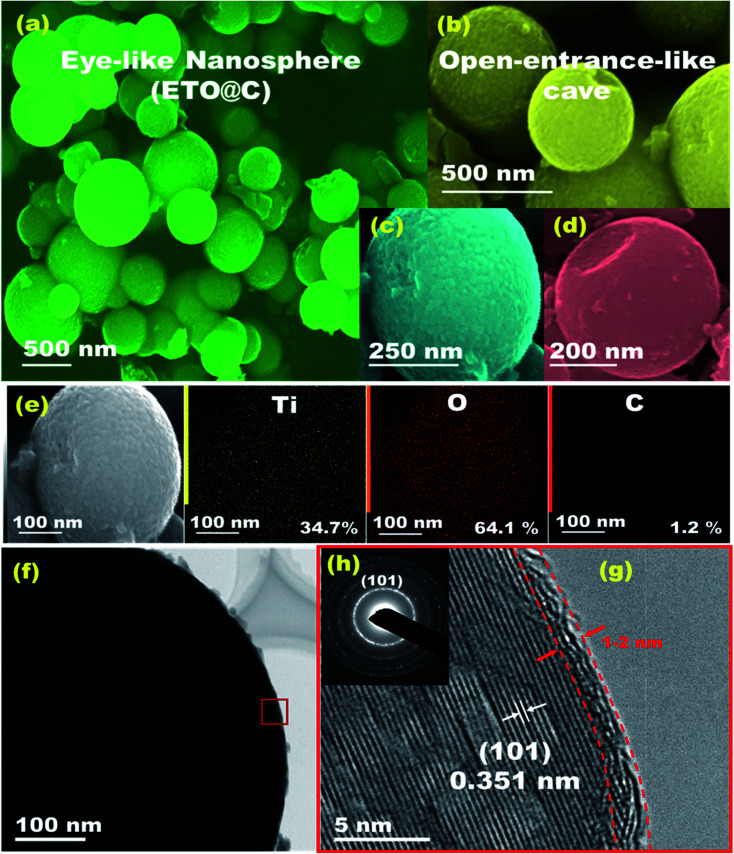
Microscopic analysis patterns based on (a–d) FE-SEM, (e) EDX, (f and g) HR-TEM, and (h) ED images of mesoscopic super-open-eye core/shell spheroid ETO@nano-C anode. (h) The selected area of the ED pattern image recorded along the [101] plane of ETO@nano-C anode geometrics.

The geometric structures of mesoscopic super-open-eye core/shell spheroids consisting of LFPO@nano-C cathodes and ETO@nano-C anodes are shown in Fig. 1, 2, S3, and S4.† Various 3D-LFPO@nano-C geometrics including SSB@nano-C, MS@nano-C, and DCS@nano-C cathode materials were examined using field emission scanning electron microscopy (FE-SEM) (Fig. 1(a–h)). The SSB@nano-C cathode material exhibited unique features of multi-diffusive open-pore systems and high-facet nanoscale cuboid protrusions that were densely distributed on the exterior spheroid surfaces. These interior/exterior accommodation/storage pockets increase the electrochemical effectiveness and fully reversible capability of LIBs. The open-mesoeye ETO@nano-C anode shows edge-loop curvature, leading to increased electronic conductivity, short electron transport distance, and fast diffusive kinetics of Li^+^ ion at the electrolyte/electrode interfaces during lithiation/delithiation processes (Fig. 2(a–e)). Among the cathode spheres, the outer sphere SSB@nano-C cuticles are wrapped by mosaic tiles oriented in six nano-[001] cuboid-exposed facets. The super-open-eye uniqueness of the SSB@nano-C core/shell spheroids offers a free space of surfaces, enabling multi-directional and multi-central dynamic diffusion efficiencies of Li^+^ ions with discharge/charge (lithiation/delithiation) cycles. The chemical composition microanalysis of energy-dispersive X-ray (EDX) spectroscopy patterns of the spheroid hierarchy with the open-mesoeyes of ETO@nano-C (anode) and 3D-LiFePO_4_@nano-C (cathode) composites (Fig. 1(f), 2(e), S3 and S4†) show evidence of inorganic–organic hybrid composites, and surface heterogeneity of electrodes. The elemental mapping of the SSB@nano-C cathode composites is homogenously distributed along the entire core/shell spheroids with Fe : P : O : C elemental ratios of 16.9 : 16.7 : 64.1 : 2.3%, respectively.

Microscopic analysis patterns based on electron diffraction (ED) and highly resolved transmission electron microscopy (HRTEM) images of the ETO@nano-C anode and 3D-LFPO@nano-C cathode composites (Fig. 1(i–k), 2(f–g), S3(d–f) and S4(e–g)†) show evidence of the formation of (i) super-open-eye core/shell spheroids in concave curvature folds, (ii) nanoscale surface roughness ripples, ridges, bumps, and undulations, (iii) atomic-scale ordering structures of LFPO@nano-C spheres, and (iv) 2–4 nm sizable feathery carbon shell layers covering the spherically exterior surfaces of SSB@nano-C, MS@nano-C, and DCS@nano-C cathodes and ETO@nano-C anode materials. These surface features allowed the fabrication of cathode/anode electrodes with high electronic conductivity and ionic diffusion dynamics, thereby facilitating the electron/Li^+^ ion transport in the super-open-mesoeye LIB model systems. The microscopic edge patterns of the SSB@nano-C, MS@nano-C, and DCS@nano-C cathode core/shell spheroids show atomic crystal phase planes with *d*-spacing values of (0.456, 1.03), (0.426, 0.38), and (0.458, 1.05) nm for the (001), (100), (−101), (−2−10), and (001), (100) planes, respectively (Fig. 1(i–k)). The ED profiles of the SSB@nano-C, MS@nano-C, and DCS@nano-C core/shell spheroids display high crystal planes along the [010] dominant facets of the orthorhombic olivine structure LFPO phase with interatomic *d*-spacing values (0.456, 1.03), (0.426, 0.38), and (0.458, 1.05) nm, respectively; see Fig. 1(l), S3(f) and S4(g),† respectively. The predominant exposure [010] *ac*-plane sites provide low surface energy sites for (i) reasonable free-space volumes for electrons/Li^+^ ion transports during charging/discharging (lithiation/delithiation) cycles, and (ii) fully reversible capability.^49–51^ The HR-TEM image of ETO@nano-C core/shell spheroids showed the formation of layers that were 1–2 nm thick of C-shell dressers along the entire mesosphere surfaces (Fig. 2(f) and (g)). The surface homogeneity of ordered dressing of nano-carbon layers creates multiple directional gates, and active surface mobility sites. In addition, the ED pattern of ETO@nano-C displays a clear pattern with a (101) plane and *d*-spacing of 0.351 nm of the anatase phase with a tetragonal (*I*4_1_/*amd* space group) structure (Fig. 2(h) and S10†).

The textural parameters and space holes of mesoscopic super-open-eye core/shell spheroids can explain the main effect of surface morphology on the creation of diffusion gateways for achieving fast kinetic charge–discharge rates, as reflected in the N_2_ adsorption–desorption isotherms (Fig. S5†). The well-ordered, crystal phase component, structural properties, thermal stability, and sustainable coating layers of C-shells along the SSB@nano-C, MS@nano-C, and DCS@nano-C cathode and ETO@nano-C anode mesoeye spheres were determined *via* X-ray diffraction (XRD), X-ray photoelectron spectrometry (XPS), thermogravimetric and Raman spectra analyses, and Fourier transform infrared (FTIR) spectra (Fig. S5–S10†).

### 3.3. Buildup configuration of half- and full-scale super-open-mesoeye LIB-cells and pouch models

**Scheme 2:**
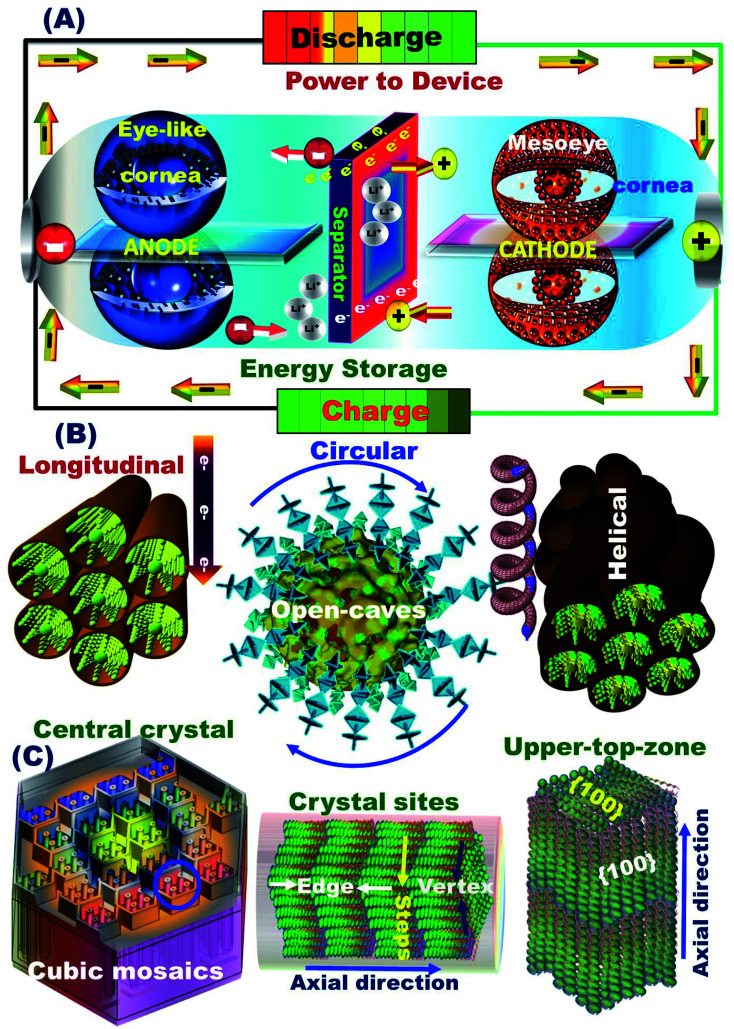
(A) Systematic design of fully reversible lithiation (discharge)/delithiation (charge) cycles of full-scale ETO@nano-C anode//SSB@nano-C cathode CR2032-coin cells. (B) Electron/Li^+^ ion transfer diffusion controls during charge/delithiation (anodic oxidation) and discharge/lithiation (cathodic reduction) cycles along super-open-eye core/shell SSB@nano-C-cathode (P-electrode) spheroids. (C) Surface topology of 3D cuboid mosaics (SSB@nano-C, cathode) along the active-site surfaces.

The structurally stable atomic-scale arrangements, mesoscopic super-open-eye core/shell spheroids, and interior uniform accommodation/storage space pockets (*i.e.*, surface mesogrooves and mesoeye entrances, and interior innumerable caves and core hollow nests) may create fully-reversible, dynamic LIB models. Our half- and full-scale super-open-mesoeye LIB-cells and pouch models feature continuous electron/Li^+^ ion diffusions and transports, dense flow rates in possible directions, and heavily inertial Li^+^ ion loads during charge–discharge reversibility cycles (Scheme 2). As practical LIB design sets, the 3D-LFPO@nano-C mesoscopically-shaped open-eye spheroid structures including SSB@nano-C, MS@nano-C, and DCS@nano-C geometrics enable the fabrication of potential cathode electrodes designed into half- and full-LIB-CR2032 designs, and large-scale pouch models. Mesoscopic engineering of ETO@nano-C and SSB@nano-C as potential N and P electrodes enables long-term cycle stability for half-cell anode and cathode LIB-CR2032 coin cells, respectively. In these variable LIB models, the buildup ETO@nano-C (anode)//SSB@nano-C (cathode) LIBs with mesoscopically structured geometrics, and broad-free-access surfaces including hollow grooves, geode caves, and entrances boost the electron/Li^+^-ion diffusivity and charge–discharge reversibility. We also fabricated dynamic pouch-type LIB models using densely ordered collar packing of CR2032-coin cell sets. This pouch-type LIB provides sustainable rate capability, remarkable areal discharge capacities, and high gravimetric and volumetric energy densities.

### 3.4. Variable half-scale 3D-LFPO@nano-C cathode-configured LIB-CR2032 designs

**Fig. 3:**
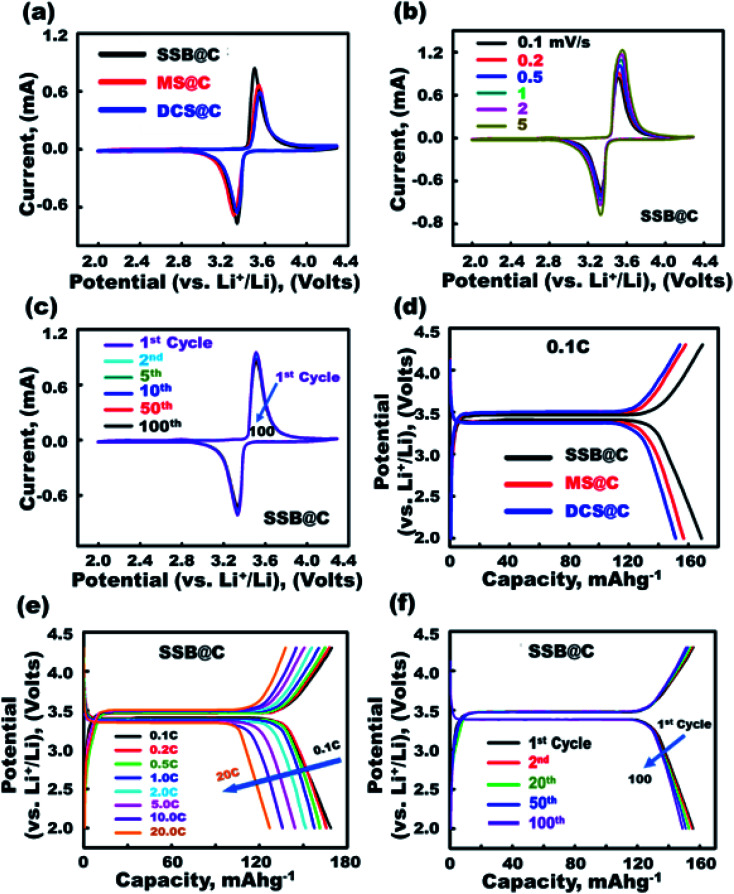
(a–c) CV profiles of the electrochemical effectiveness of half-cell cathode SSB@nano-C, MS@nano-C, and DCS@nano-C LIB-CR2032 designs. (d) The 1^st^ discharge capacity of half-cell cathode SSB@nano-C, MS@nano-C, and DCS@nano-C LIB-CR2032 designs. (e and f) 1^st^ discharge capacity of an SSB@nano-C cathode LIB at various C-rates from 0.1–20C and at different 1^st^ to 100^th^ cycles and 1.0C.

**Fig. 4:**
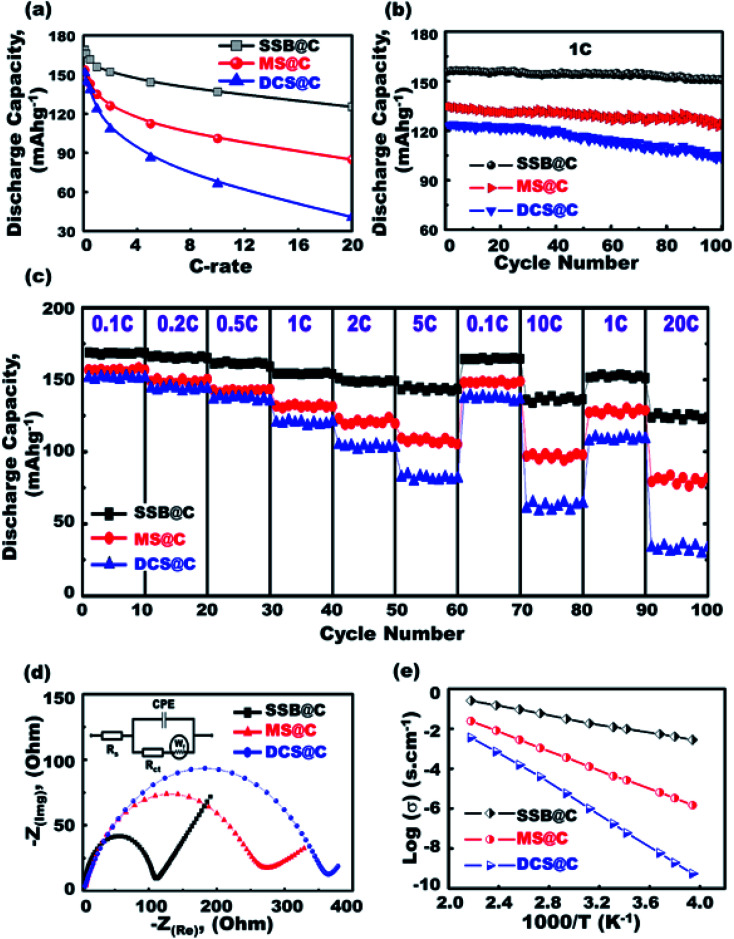
(a–e) Electrochemical performances of SSB@nano-C, MS@nano-C, and DCS@nano-C cathode LIB-CR2032 coin-cell models. (a) 1^st^ discharge specific capacity at 0.1–20C. (b) Cycling performance stability at rate of 1.0C for 100 cycles. (c) Capability performance rates at 2.0–4.3 V and 0.1–20C. (d) The EIS analysis (the inset shows the equivalent circuit diagram). (e) The electrical conductivity-dependent temperature of half-cell cathodes.

Super-open-eye half-scale 3D-LFPO@nano-C cathode LIB-CR2032 configuration designs with variable cathode geometrics include SSB@nano-C, MS@nano-C, and DCS@nano-C P electrodes. Cyclic voltammetry (CV) profiles were used to evaluate the electrochemical features of SSB@nano-C, MS@nano-C, and DCS@nano-C cathode half-cell LIBs (Fig. 3 and 4).

The integral effects of structurally stable surface topologies, atomic-scale crystal planes, mesoscopic super-open-eye core/shell spheroids, and grooves as accommodation/storage space pockets on the effective 3D-LFPO@nano-C cathode LIB-CR2032 designs were studied, as shown in Fig. 3 and 4. The CV measurements for all designed cathodic electrode materials were performed at the C-rate of 0.1, and at voltage window of 2.0–4.3 V (Fig. 3(a)). Based on the obtained CV results, the main reduction/oxidation profile peaks were found at 3.33/3.5 V, 3.3/3.55 V, and 3.33/3.56 V for super-open-mesoeye SSB@nano-C, MS@nano-C, and DCS@nano-C cathode LIB-CR2032 designs, respectively. To explore the effect scan rates on the super-open-mesoeye cathode LIB-CR2032 designs, the CV measurements of the cathode SSB@nano-C geometrics were performed at different C rates in the 0.1–5C range and 2.0–4.3 V; see Fig. 3(b). With increasing scan rate (*i.e.*, from 0.1–5.0C), the potential for oxidation peaks increases, and the potential reduction values decrease. Additionally, there is a clear increase in the current values of all oxidation (delithiation)/reduction (lithiation) profile peaks associated with the C-rate enhancement. The cyclic stability of the SSB@nano-C cathode half-cell was tested at the 1^st^ to 100^th^ cycle and C-rate ranges. The highly symmetric peaks of oxidation (delithiation)/reduction (lithiation) Fe^3+^/Fe^4+^ (LiFePO_4_/FePO_4_) profiles for the SSB@nano-C cathode were observed at 3.33/3.5 V. This profile reveals that the electrochemical reaction of the tested SSB@nano-C cathode is fully reversible during the lithiation/delithiation process.^52,53^ The effect of variable half-cell cathode P electrode geometrics on the electrochemical capacity performance was investigated during the first lithiation/delithiation cycle process at 2.0–4.3 V range and 0.1C (Fig. 3(d)). The obtained results of the charge–discharge cycling performance profiles of half-cell cathode LIBs displayed similar behavior for SSB@nano-C, MS@nano-C, and DCS@nano-C half-cell cathode LIBs. This finding indicates that the reversibility and cycling performance is outstanding. In addition, the discharge capacity of the different cathodic electrodes indicates that the discharge capacity of the SSB@nano-C cathode material is higher than the other tested MS@nano-C and DCS@nano-C electrodes. The 1^st^ discharge capacity at 0.1C for the SSB@nano-C-, MS@nano-C-, and DCS@nano-C cathodes was found to be 168.8, 156.8, and 151.4 mA h g^−1^, respectively. The high performance of the SSB@nano-C half-cell cathode was associated with the outer wrapping of sphere cuticles by mosaic tiles oriented in 6-facet nano-[001] cuboid-exposed facets. The well-dispersed protrusions with cubically exposed active facets along the SSB@nano-C sphere surfaces offer broad and large free surfaces and volume spaces for a suitable Li^+^ ion diffusion. Further studies clarified the effect of the C-rate range (0.1–20C), and cycle numbers on the (i) 1^st^ cycle discharge capacity and (ii) charge/discharge stability performance, respectively (Fig. 3(e) and (f)). The experimental sets were conducted using SSB@nano-C electrodes configured for a half-scale cathode LIB-CR2032 coin-cell, Fig. 3(e). The finding of a super-open-mesoeye SSB@nano-C cathode indicates its outstanding discharge capacity behavior by increasing the C-rate (0.1–20C), and various cycle numbers, Fig. 3(e and f). Overall, the charging/discharging profiles of the half-scale SSB@nano-C LIB-CR2032 coin-cell reveal the long-term cycling performance of lithiation/delithiation (discharge/charge) processes.

To confirm the effective half-scale super-open-mesoeye 3D-LFPO@nano-C cathode geometrics on the first discharge capacities, we investigated the effect of variable SSB@nano-C-, MS@nano-C-, and DCS@nano-C cathode geometrics on (i) 1^st^ cycle discharge capacity, and (ii) discharge stability performance at various C-rates and multiple cycle numbers in the 1^st^ to 100^th^ range (Fig. 4(a) and (b)). The discharge capacity performance was decreased by increasing the C-rate for all half-cell 3DLFPO@nano-C cathode electrodes, as shown in Fig. 4(a). The SSB@nano-C half-scale cathode LIB-CR2032 coin-cell exhibited high performances among other MS@nano-C and DCS@nano-C cathode geometrics. The capacity performance of the tested super-open-mesoeye cathodes decreases in this order: SSB@nano-C > MS@nano-C > DCS@nano-C. The cycling performance profile (Fig. 4(b)) confirmed the retention superiority of all designed 3D-LFPO@nano-C half-cell cathodes. The SSB@nano-C half-scale cathode LIB-CR2032 coin-cells exhibited superiority capacity, and the retention of the cathode amongst other electrodes is presented in Fig. 4(b). This finding shows that the retention of discharge capacities of SSB@nano-C, MS@nano-C, and DCS@nano-C half-scale cathode LIB-CR2032 coin-cells after 100 cycles is 96.7%, 92.5%, and 83.2%, respectively. The sustainability of the high discharge capacity after a number of cycles indicates the excellent electrochemical lithiation/delithiation reversibility.

A set of experiments evaluating the cycling performance of SSB@nano-C, MS@nano-C, and DCS@nano-C half-scale cathode LIB-CR2032 coin-cells at 0.1–20C, 2.0–4.3 V, and 25 °C was performed to investigate the fully reversible capability rate performance (Fig. 4(c)). The specific capacity performances of half-scale 3D-LFPO@nano-C cathode LIB-CR2032 designs were verified at C-rate sequence patterns of 0.1, 0.2, 0.5, 1, 2, and 5C. The specific capacity pattern returned to 0.1 and 10C, and then returned to 1 and 20C. The variable half-scale cathode geometrics of the 3D-LFPO@nano-C LIB-CR2032 designs showed outstanding retention rates for discharge specific capacities (mA h g^−1^) at C-rate patterns of 0.1, 0.2, 0.5, 1.0, 2.0, and 5.0C, and at different cycling numbers from the 1^st^ to 60^th^ cycles. Fig. 4(c) also indicates the effective retention behaviors as follows:

(i) The specific capacity performance is decreased by increasing the C-rate for all half-cell cathode electrodes,

(ii) The discharge capacities for all cathode geometrics at the reduced rate pattern, for example, from 0.5C to 0.1C and also from 10C to 1C, are fully recovered, indicating a high rate capability of cathodes,

(iii) the SSB@nano-C half-scale cathode LIB-CR2032 coin-cells demonstrated a remarkable rate capability amongst other electrodes, and the sequence order is as follows: SSB@nano-C > MS@nano-C > DCS@nano-C geometrics, as shown in Fig. 4(c), and

(iv) The discharge reversible capacities at 20C after the 100^th^ cycle for the SSB@nano-C and MS@nano-C half-scale cathode LIB-CR2032 coin-cells were 123.7 and 81.8 mA h g^−1^, respectively, compared to the discharge reversible capacity of 33.6 mA h g^−1^ for DCS@nano-C.

A setup of EIS electrochemical impedance spectroscopy patterns for half-scale 3D-LFPO@nano-C cathode LIB cells was investigated, and the equivalent circuit is shown in the inset in Fig. 4(d).^49,54,55^ The resultant semicircles of Nyquist plots and charge transfer resistance (*R*_ct_) indicate the functional activity of the cathode geometrics. In this regard, the SSB@nano-C half-scale cathode LIB-CR2032 coin-cell showed rapid electron/Li^+^ ion diffusion kinetics, low-resistance load surfaces, and short transport distance for electron movements compared with other cathodes, as evidenced from the small semicircle diameter and *R*_ct_ value. Maintaining the battery sustainability and effectiveness at different temperatures is crucial. The electrical conductivity of the SSB@nano-C, MS@nano-C, and DCS@nano-C cathode electrodes designed for half-scale cathode LIB-CR2032 coin-cells was examined at a wide range of temperatures (250 to 455 K), as shown in Fig. 4(e). Fig. 4(e) exhibits evidence that (i) all cathodes maintained high conductivity at 298 K, (ii) the surface sustainability of all electrodes decreased by increasing the environmental temperature, and (iii) the SSB@nano-C half-scale cathode LIB-CR2032 coin-cell revealed superior sustainability of dynamic electron mobility and then electrical conductivity performance against high temperature treatment.

Overall, our findings indicate that the SSB@nano-C electrode geometrics with super-open-mesoeye core/shell spheroids, a bundle of upward/outward or convex/concave curvature folds, and uniform distribution of 6-facet cuboid-capped gradients along the exterior spheroid surfaces lead to LIB designs with facile Li^+^-ion diffusion, minimized electron transport distance, suitable storage and accommodation, and high rate capability and energy density (see ESI S11 and S12†).

### 3.5. Super-open-eye half-scale ETO@nano-C anode LIB-CR2032 configuration designs

**Fig. 5:**
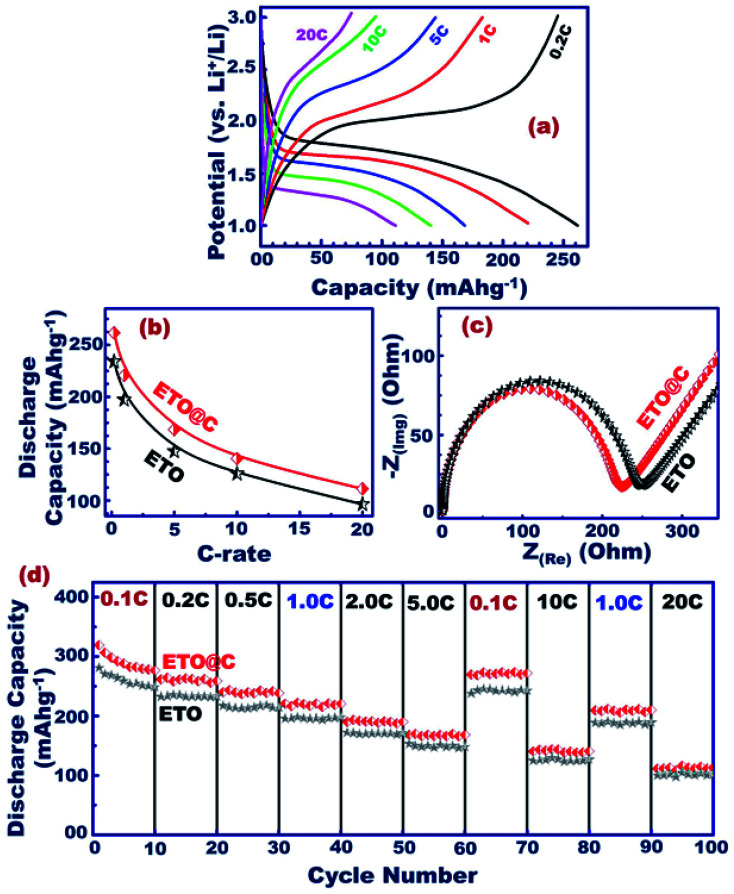
(a–d) The electrochemical studies of the pristine ETO and ETO@nano-C anodic half-scale LIB-CR2032 coin-cell. (a) Charge–discharge voltage analysis at first cycle with multi C-rates (0.2–20C). (b) 1^st^ discharge capacity of ETO@nano-C and pristine ETO anode geometrics at various C-rates upon discharge. (c) EIS profile of ETO@nano-C and pristine ETO anode geometrics. (d) Capability performance rates at 1.0–3.0 V and 0.1–20C.

The engineering of mesoscopic super-open-eye core/shell ETO@nano-C spheroids as a potential anode electrode leads to formulation of long-term cycle stability for LIB-CR2032 coin cells with high charge–discharge capacity and capability performance rate (Fig. 5 and S1†). The behavior of the specific charging/discharging capacity of half-scale ETO@nano-C anode LIB-CR2032 designs at 0.2–20C and 1.0–3.0 V was investigated. The discharge curve reveals a drastic decrease in the capacity from 3.0 to 1.7 V due to the insertion of Li^+^ ion into the ETO@nano-C anode through super-open-mesoeye entrances. The gradual decrease to 1.0 V indicates a fully dynamic lithiation process into ETO@nano-C electrode spheroids. In turn, the increase step in the charging profile at a voltage range from 1.0 to 1.9 V occurred because of delithiation modes. The gradual and fast increase in charge pattern indicates the large amount of lithiation of Li^+^ ion through the interfacial open-eye storage accommodation.

We studied the effect of various C-rates on the 1^st^ cycle discharging capacity of ETO@nano-C and pristine ETO anode geometrics (Fig. 5(b)). Both anodes showed a similar reduction in their discharge capacity profile with increasing C-rates. In turn, the outstanding specific capacity of the ETO@nano-C half-cell at all C-rates was evident compared with the ETO anode. The Nyquist analysis of the ETO@nano-C anodic half-scale LIB-CR2032 coin-cell shows a smaller semicircle diameter than that of the pristine ETO anode. This result indicates minimum *R*_ct_ and transfer resistance values, and rapid diffusion dynamics of electrons/Li^+^ ions along broad free-access surfaces of the large-open-eye ETO@nano-C anode (Fig. 5(c)).

The cycling retention of the discharge capacity performance of the ETO@nano-C and ETO@nanocore anode half-scale LIB-CR2032 coin-cells at different 0.1–20C rate ranges is an important key for investigating the rate capability of the LIB design. In such experimental sets, the current rate was recorded with 10 cycles at each scan C-rate, at 1.0–3.0 V, and with 100 cycles (Fig. 5(c)). The discharge profile of both anodes indicates the inverse relationship between the discharge capacity and C-rates. For instance, the capacity of ETO@nano-C significantly decreased from 319.2 to 113.1 mA h g^−1^ at 0.1C and 20C at the 1^st^ cycle, respectively. In addition, the decorating of ETO surfaces with 3–5 nm carbon shells leads to the superior cycling performance of the ETO (core)@nano-C (shell) anode compared with the ETO anode. The C-rate/discharging pattern at 0.5C returns to C-rates of 0.1 and 10C, and continuously returns to rates of 1C and 20C, respectively (Fig. 5(d)). The slight change in the backup discharge capacity indicates the stability of the anode half-cell and its high rate capability. The full recovery of the anode discharge capacity at reduced or elevated C-rate patterns indicates the high rate capability, and the fast kinetic Li^+^-ion diffusion/transport/accommodation effectiveness.

### 3.6. Full-scale ETO@nano-C anode//SSB@nano-C cathode CR2032-coin cells and pouch LIB models

We selected mesoscopic core–shell ETO@nano-C anode//SSB@nano-C cathode electrodes for potential full-LIB-CR2032 configuration designs (see ESI S1 and S2†). Both ETO@nano-C anode and SSB@nano-C cathode electrodes enable built-in LIBs with excellent charge/discharge capacity and energy density performances, as well as outstanding cycling stability and trade-off factors. In this full-scale CR2032-coin LIB cell, an optimal tradeoff function of full-cell LIBs can be achieved *via* special control of the mass balancing ratios of the SSB@nano-C cathode (P electrode) and ETO@nano-C anode (N electrode) (*i.e.*, the balancing (P/N)_Cap_ ratio ≈1.0 : 1.07–1.2). The key design configuration is achieved at a specific (P : N)_Cap_ ratio ≈ 1.0 : 1.1; see ESI S2.† Furthermore, the specific energy density of the super-open-eye full-cell ETO@nano-C//SSB@nano-C LIB is 333.99 (≅334) W h kg^−1^. The effective mass fraction of the P electrode (SSB@nano-C) in the CR2032-coin full-cell LIB model has been designed to be approximately 49.6%, enabling the formulation of a specific energy density battery at full-cell LIB mode of approximately 165.66 W h kg^−1^.

**Fig. 6:**
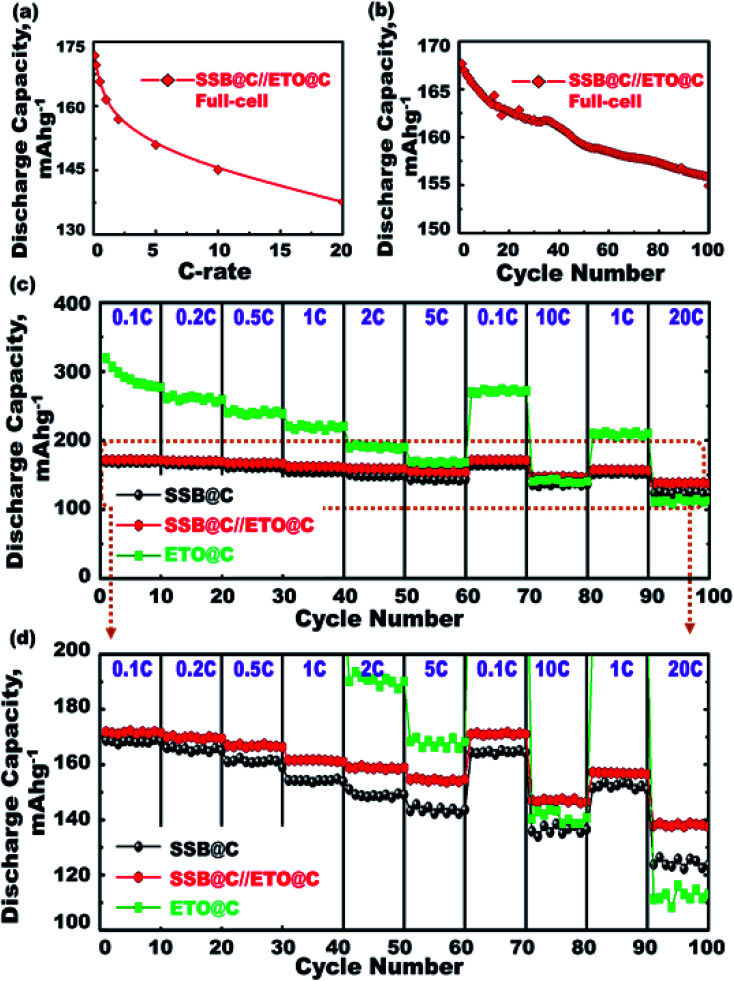
(a–d) The electrochemical performances of ETO@nano-C-anode//SSB@nano-C-cathode CR2032-coin full-cell LIB models within potential range of 0.8–3.5 V. (a) The specific discharging capacity as a function of C-rate changes (*i.e.*, 0.1–20C range). (b) The specific discharging capacity performance as a function of cycle numbers. (c and d) The rate capability analysis and its enlarged profiles at various C-rates (0.1–20C).

Fig. 6(a) shows the C-rate dependence in the range of 0.1–20C for the 1^st^ cycle discharge capacity at a potential range of 0.8–3.5 V for the ETO@nano-C anode//SSB@nano-C cathode CR2032-coin full-cell. The discharge capacity is decreased by increasing the C-rate. For example, the discharge capacity is decreased from 172 mA h g^−1^ at 0.1C to 137 mA h g^−1^ at 20C. Fig. 6(b) shows the discharge stability performance of ETO@nano-C anode//SSB@nano-C cathode CR2032-coin cells at cycle numbers of the 1^st^ to 100^th^ range. The full-cell discharge capacity is retained by 92.4% of its first cycle at 1C and after 100 cycles, indicating the excellent charge–discharge reversibility process. This finding indicates the retention of the electrode's electronic conductivity and excellent electron/Li^+^ ion transfer kinetics during charge/delithiation (anodic oxidation) and discharge/lithiation (cathodic reduction) cycles.

The super-open-eye LIB rate capability performance of full-scale ETO@nano-C//SSB@nano-C CR2032-coin cells was studied at various cycle numbers of the 1^st^ to 100^th^ range, C-rates of 0.1–20C, and voltage range of 0.8–3.5 V. Each sweep C-rate dependence profile was recorded with 10 cycles and up to 100 cycles, and at 25 °C (Fig. 6(c and d)). The decrease in the specific capacity of full-scale ETO@nano-C//SSB@nano-C CR2032-coin cell LIBs with increases in the C-rate is evident. The discharge capacity of the full-scale ETO@nano-C//SSB@nano-C CR2032-coin cell LIB was retained at 1C (40 cycles) and 5C (60 cycles), and returned to 10C (80 cycles) and 20C (100 cycles) with retention ratios of its 1^st^ discharge capacity of 99.65%, 99.03%, 96.8%, and 98.7% at these specific rates, respectively. The effective rate capability leads to the facile reversibility of lithiation (discharge/reduction)//delithiation (charge/oxidation) processes at SSB@nano-C cathode/ETO@nano-C anode surface electrodes (*i.e.*, Li^+^-insertion/Li^+^-extraction mechanism), respectively. The maintaining of broad free-access and large-open-eye like gate-in-transport surfaces along the SSB@nano-C cathode/ETO@nano-C anode electrodes within cycles are key factors that are built into LIBs with outstanding rate capability, excellent charge/discharge capacity and energy density performances, and fully reversible and cycled dynamics (Scheme 2).

**Fig. 7:**
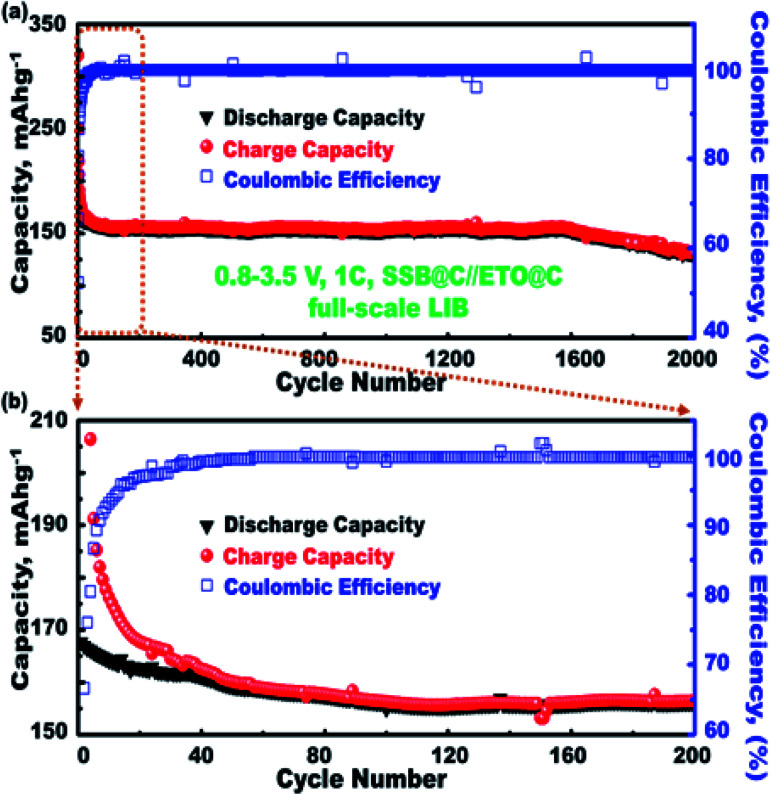
(a) The outstanding cycling stability and coulombic effectiveness for full-scale SSB@nano-C//ETO@nano-C LIB models, at 1C and multiple cycles (1^st^ to 2000^th^ cycle). (a) The electrochemical performance measured at 0.8–3.5 V and 25 °C, and (b) the magnification of the first 200 cycles.

The outstanding cycling stability of mesoscopic open-eye spheroids in full-scale ETO@nano-C//SSB@nano-C LIB-CR2032 was designed as a function of charging–discharging specific capacity, and coulombic performance effectiveness was investigated (Fig. 7(a and b)). The charging–discharging cycling profiles (*i.e.*, 1^st^ to 2000^th^ cycle) of the ETO@nano-C//SSB@nano-C CR2032-coin cell LIB were performed in the potential range of 0.8–3.5 V, 1C, and at 25 °C. Overall, the designed ETO@nano-C//SSB@nano-C CR2032-coin cell LIB exhibited superiority with a retention capacity of 77.8% of the 1^st^ cycle, discharge capacity of 168.69 mA h g^−1^ after multiple cycling (*i.e.*, 1^st^ to 2000^th^ cycles), and efficient coulombic performance of approximately 99.6% at 0.1C. These results indicate long-period cycling stability in terms of a remarkable charge–discharge cycle capacity performance, and outstanding sustainability in coulombic effectiveness over the 2000^th^ cycle (Fig. 7).

The superior electrochemical performance of the designed ETO@nano-C//SSB@nano-C CR2032-coin cell LIB confirms the effectiveness of the P and N electrode geometrics and surface topologies with nanoscale cuboid protrusions, free surface volume space, multidirectional and multicentral dimensionality entrances, and interior uniform accommodation/storage space pockets (*i.e.*, surface mesogrooves and mesoeye entrances, and innumerable interior caves and core hollow nests) (Scheme 2). The super-open-eye full-cell LIBs offer large gate-in transport and out/in, up/down, and circular/curvy movement folds, enabling innumerable caves to shelter the electron/Li^+^ ions during storage charging/discharging power density, with fully reversible capability (Scheme 2).

### 3.7. Large-scale, collar packing of CR2032-coin cell sets in the pouch LIB model

For practical and large-scale usage of LIBs in EVs, the fabrication of super-open-eye pouch LIB models with specific 3D dimensions of 35 mm (width), 55 mm (length), and 2.5–3 mm (thickness) was tested by using multiple rolls of ETO@nano-C//SSB@nano-C CR2032-coin cells. Well-organized coin cells were packed in a collar fashion to form pouch LIB models (Scheme 1). In the cell package, the dense layers of the ETO@nano-C anode (5 layers/10 sides)//SSB@nano-C cathode (6 layers/10 sides) can be oriented in the coin cells and contiguously connected into a series to configure pouch-type LIBs. The volumetric energy density (*i.e.*, battery capacity in volume) of the super-open-mesoeye pouch LIB configuration is 215.14 W h l^−1^ (see ESI S3†). Remarkable free-space surface storage, areal discharge capacity, and volumetric energy density exist in a full-scale packing of SSB@nano-C//ETO@nano-C LIB cells in a pouch model system, which can be a force-driven LIB design for EV applications.

## 4. Conclusions

Powerful half- and full-scale anode//cathode LIB-CR2032 and pouch-type models were designed using super-open-eye spherule anode/cathode electrode tectonics. Variable half-, full-, and large-scale LIB super-open-mesoeye geometric models were tailored along ETO@nano-C anodic N electrodes, as well as a variety of 3D-LFPO@nano-C projections such as SSB@nano-C, MS@nano-C, and DCS@nano-C cathodic P electrodes. The structurally stable anode/cathode electrodes with unique atomic-scale organization, mesoscopically shaped open-eye spheroids, and interior uniform accommodation/storage space pockets (*i.e.*, surface mesogrooves and mesoeye entrances, and interior innumerable caves and core hollow nests) may create fully reversible, dynamic LIB models. The N and P electrode configuration for half- and full-scale super-open-mesoeye LIB cells and pouch models features continuous electron/Li^+^ ion diffusion and transport, dense flow rates in possible directions, and heavily inertial Li^+^ ion loads during charge–discharge reversibility cycles. As a result, the proposed 3D super-scalable SSB@nano-C//ETO@nano-C large-scale LIB-CR2032-coin cell models may lead to high cycling performance, non-prescriptive stability cycle usages, and dense and broad free-access surfaces. Our mesoscopic open-eye spheroid full-LIB-CR2032 configuration models retain 77.8% of the 1st cycle discharge specific capacity of 168.69 mA h g^−1^ after multiple cycling (*i.e.*, 1–2000 cycles) and efficient coulombic performance of approximately 99.6% at 0.1C, thereby facilitating lithiation/delithiation processes. The super-open-eye ETO@nano-C//SSB@nano-C LIB-CR2032-coin cell scale provides a high specific energy density battery of approximately 165.66 W h kg^−1^ at a C-rate of 1.0C, which transcends the qualification for a LIB that can support long-range driving of EVs. A successful design of dynamic pouch-type LIB models is modulated in a collar packing pattern of the P and N electrode LIB-CR2032-coin cell. Our full-pouch package LIB models provide high areal discharge capacities and remarkable gravimetric and volumetric energy densities, as well as affordable off/on free-space storage stability of pouch-type LIBs.

## Conflicts of interest

There are no conflicts to declare.

## Supplementary Material

NA-002-D0NA00203H-s001
