# Supplementary material for: Mesoscopic open-eye core–shell spheroid carved anode/cathode electrodes for fully reversible and dynamic lithium-ion battery models
Source: Nanoscale Adv. 2020 Jul 9;2(8):3525–41. doi: 10.1039/d0na00203h (PMC9418016; doi:10.1039/d0na00203h)
Supplement: NA-002-D0NA00203H-s001 [file NA-002-D0NA00203H-s001.pdf]

Electronic Supplementary Material (ESI) for Nanoscale Advances.
This journal is © The Royal Society of Chemistry 2020

Supporting Information:

Mesoscopic Open-eye Core-Shell Spheroids Carved Anode/Cathode Electrodes for fully-reversible and dynamic LIB models

H. Khalifa, [a,1] S.A. El-Safty,*[a] A. Reda, [a,2] A. Eid [a], A. Elmarakbi[b], M.A. Shenashen [a,*]

[a] National Institute for Materials Science (NIMS), Sengen 1-2-1, Tsukuba, Ibaraki 305-0047, Japan.

[b] Department of Mechanical & Construction Engineering, Faculty of Engineering and Environment, Northumbria University, Newcastle upon Tyne, NE1 8ST, UK.

E-mail: sherif.elsafty@nims.go.jp; SHENASHEN.Mohameda@nims.go.jp

Webpage: https://samurai.nims.go.jp/profiles/sherif_elsafty

[1] Current address: Department of Physics, Faculty of Science, Damanhur University, Damanhur, Egypt

[2] Current address: Composite Lab, Advanced Materials Division, Central Metallurgical R&D Institute, Helwan, 11421, Egypt

S1. Experimental sets of fabrication of anode/cathode materials, electrodes and LIB cells

i. Chemicals used for fabrication of spheroid materials and their anode/cathode electrodes and coin cells

All chemicals used for fabrication of spheroid materials and their anode/cathode electrodes and coin cells were used as received without additional purification. Iron (III) chloride, iron (III) sulfate hydrate, iron (III) nitrate nonhydrate, titanium(IV) isopropoxide, acetonitrile, ammonia solution, ethanol (C_2H_5OH), ethylene carbonate ($(CH_2O)_2CO$), diethyl carbonate ($C_5H_{10}O_3$), lithium hexafluoro-phosphate ($LiPF_6$), carbon black, and polyvinylidene fluoride (PVDF) are supplied from Sigma–Aldrich Company, Ltd., USA. Phosphoric acid and N-methyl-2-pyrrolidone (NMP) are obtained from Tokyo Chemical Industry (TCI) Company, Ltd., Tokyo, Japan. Ethylene glycol ($C_2H_6O_2$) and lithium sulfate are obtained from Nacali Tesque Company, Ltd., Kyoto, Japan.

ii. ***Characterization of spheroid materials and their anode/cathode electrodes***

Various techniques and tools were used to confirm and clarify the composition, structure and morphology of the mesoscopic supper-open-eye core/shell spheroids of TiO_2- and $LiFePO_4$-wrapped nanocarbon carved anode/cathode electrodes. X-ray diffraction (XRD) patters are recorded by using Bruker D8 Advance X-ray diffractometer. Microscopic patterns are recorded by using (FE-SEM) field emission scanning electron microscopy (Jeol JSM-Model 7000F, JEOL Ltd). FE-SEM is operated at 20 kV to investigate the details of mesoscopic supper-open-eye core/shell spheroids of TiO_2- and $LiFePO_4$-wrapped nanocarbon carved anode/cathode electrodes. Microscopic analysis patterns based on electron diffraction (ED) and highly-resolved transmission electron microscopy (HRTEM) images are performed to investigate the designed nanostructure anode and cathode materials (JEOL 2100F, JEOL Ltd). Moreover, BELSORP36 analyzer has been used for the N_2 adsorption–desorption isotherms analysis to investigate the surface parameters such as the surface area and pore size structure. The thermal stability of the prepared mesoscopic supper-open-eye core/shell spheroids of TiO_2- and $LiFePO_4$-wrapped nanocarbon carved anode/cathode electrode composites are analyzed using thermo-gravimetric and differential scanning calorimetry (TG/DSC) instrument TG/DSC-60 (Shimadzu, Japan) with temperature ramping 10 ^{o}C/min. Structural analysis using (XPS) X-ray photoelectron spectroscopy (PHI Quantera SXM (ULVAC-PHI) instrument is investigated by using Perkin–Elmer Co., USA. Raman spectroscopy is also investigated by using HR Micro Raman spectrometer, Horiba, Jobin Yvon. Fourier transform infrared spectroscopy (FTIR) is recorded by ATR-FTIR, Spectrum 100, Perkin-Elmer, Inc., USA.

iii. Control design of mesoscopic open-eye anode-/cathode-electrodes and CR2032-coin LIBs

All consumed chemicals during the fabrication of mesoscopic open-eye anode-/cathode-electrodes and half-, full-, and large-scale CR2032-coin LIBs have been used without further purification. The fabrication process of mesoscopic open-eye anode-/cathode-electrodes and cell design has been carried out under specific conditions in a glovebox under pure Ar-gas. The Li foil is used as a reference and counter electrode for all anode-/cathode- CR2032-coin LIB half-cells

(ETO@nano-C anode, and SSB@nano-C, MS@nano-C and DCS@nano-C cathodes) to control the electrochemical performances. In turn, we designed SSB@nano-C//ETO@nano-C anode//cathode full-cell CR2032-coin LIB formulations for electrochemical measurements of full-scale LIB-EV model system (Figure S1).

As a liquid electrolyte solution, we used a $LiPF_6$ conductive salt (1 M), which is dissolving in $(CH_2O)_2CO$, ethylene carbonate/ $C_5H_{10}O_3$, diethyl carbonate mixture with 1:1 v/v ratio. To engineer the P- and N-working electrodes by using mesoscopic 3D-LFPO@nano-C cathodic materials, and ETO@nano-C anode material, we used a mixture of the active cathode or anode materials: carbon-black: PVDF with equivalent mass fraction ratio of 0.75: 0.15: 0.1, respectively. The prepared mixture is mixed with stirring for 1 h in a rational amount of NMP. Then the prepared slurry mixtures were cast into 10 μm-aluminum (Al) and 8 μm-cupper (Cu) foils, and dried under vacuum condition (12 h/80°C). The active materials' loading mass of cathode and anode electrodes are 14.87 and 6.99 mg/cm^2, respectively.

It is important to note that the dried electrode films were compressed between the double rollers for the following key facts; (i) enhancement of the packing density, (ii) reduction of the voids and space vicinities along film surfaces, and (iii) ensuring the intimate contact of the super-open-mesoeye materials onto electrode surfaces and their electric current collector. Circular perforated electrodes (i.e., 16 mm / diameters for Li-chip reference or counter electrodes, and super-open-mesoeye anode/cathode working electrodes, respectively), and 20 mm for porous-membrane separators are prepared and combined in half-/full- scale CR2032-type coin cells using crimper machine for cell pressing.

The electrochemical performances of the designed LIB-coin-cells were tested by using galvanostatic charge/discharge merits (using multichannel battery system, LAND CT2001A, Wuhan, China). Cyclic voltammetry analysis (CV) is recorded by using CHI 660c electrochemical workstation). We used Zennium/ZAHNER-Elektrik GmbH & CoKG to measure the electrochemical impedance spectroscopy (EIS). In addition, ZS-102 tap density meter was used measure the tap density of the electrodes. All the electrochemical measurements of half- and full-scale LIB cells are carried out at 25 ^{o}C.

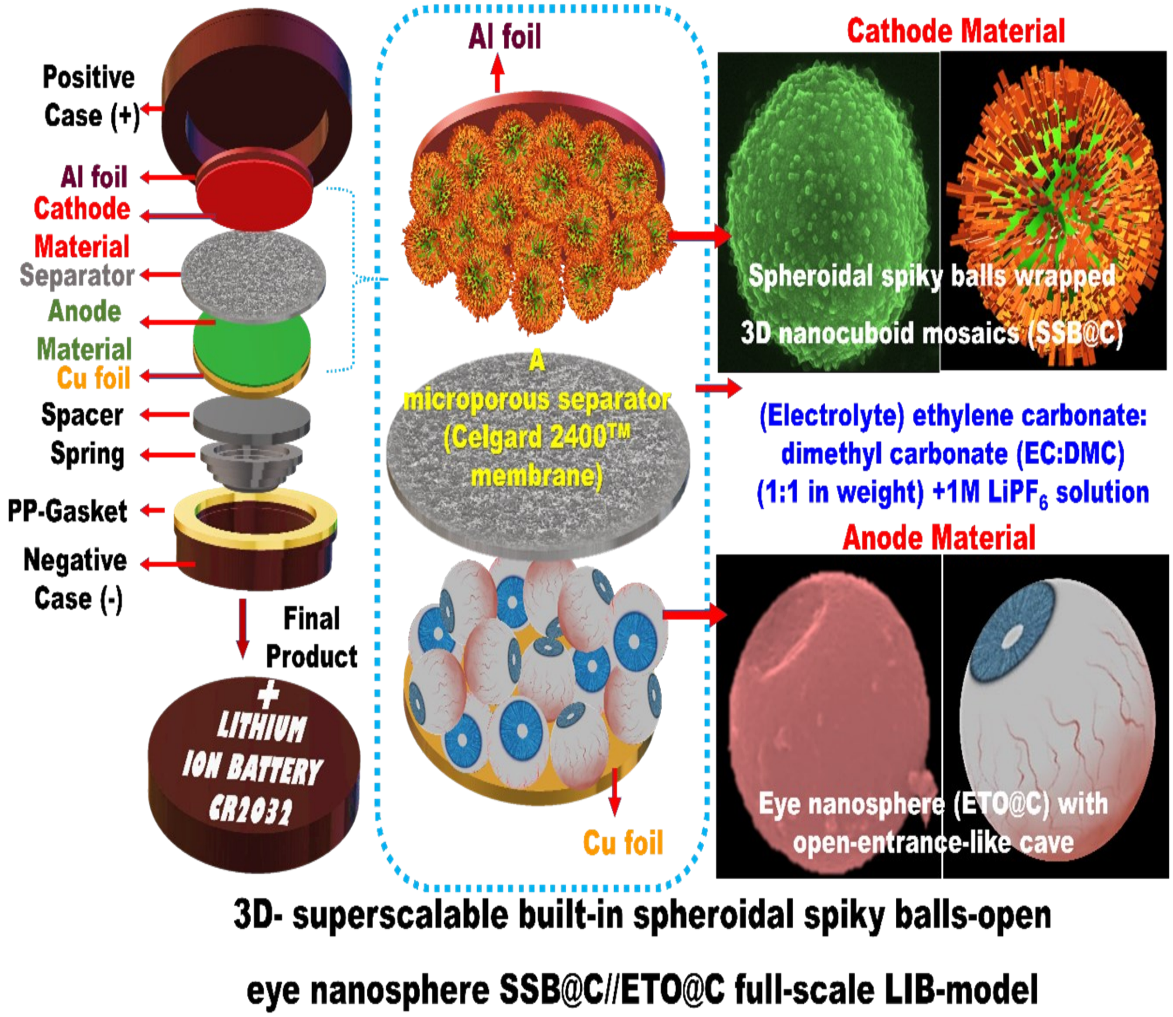

Fig. S1. Schematic design of the CR2032-type coin cell assembly that illustrate the formulation of heterogeneous built-in spheroidal spiky balls wrapped 3D nanocuboid mosaics (SSB@nano-C) cathode // open eye nanosphere with open-entrance-like cave (ETO@nano-C) anode designated in coin-cell CR2032-type full-scale LIB-model.

S2. Design parameters of 3D-LFPO@nano-C cathode and ETO@nano-C anode CR2032-coin cells

(i) P-, and N-electrodes mass balancing capacity ratio (.i.e., the balancing $(P/N)_{Cap}$) in CR2032-coin cells

The specific mass value of P- and N-electrode materials presented in the ETO@nano-C-anode and 3D-LFPO@nano-C cathode designed CR2032-coin LIB formulations is key to control the electrochemical performance features including energy density, cell capacity, and safety factors. One of electrochemical optimization and safety fabrication factor are firmly related with P / N mass proportion ratio. For instance, the mas loading affect the formation of lithium plating/deposition along anode surfaces during the charge or delithiation procedure.

The safety issue and maintaining high specific energy have a great concern tradeoff factor during the configuration of LIB-CR2032-coin-cells. Thus, controlling of the balancing $(P/N)_{Cap}$ ratio is the key design of ETO@nano-C//SSB@nano-C full-scale LIB-CR2032-coin-cells configuration and also the ordering sets of stacked-layers of ETO@nano-C LIB-CR2032-coin anode (N-electrode), and SSB@nano-C LIB-CR2032-coin cathode (P-electrode) packing into pouch models. To avoid the negative impacts and shortcomings of lithium plating mechanism during the multiple charge process, which is largely a deteriorating aging of LIB design and effectively affect the large-scale LIB safety manufacture, we fabricate the ETO@nano-C layers along the anode surfaces with $(P: N)_{Cap}$ ratio of 1: >1. A slight increase in the overall mass loading capacity of the ETO@nano-C anode is furthermore required for both furtherance safety and maintaining high specific energy storage [1-10]. We also kept, to high extent, the balancing $(P: N)_{Cap}$ ratio of 1:1 to obtain full-scale LIBs with high specific energy storage.

Per of our experimental sets, the balancing capacity of 3D-LFPO@nano-C cathode and ETO@nano-C anode with $(P: N)_{Cap}$ ratio ≈1: 1.07–1.2, to obtain an optimal tradeoff relationship. Therefore, we fabricated our ETO@nano-C//SSB@nano-C full-scale LIB-CR2032-coin-cells configuration and scalable pouch LIB-models with optimization of the mass loading capacity of 3D-LFPO@nano-C cathode and ETO@nano-C anode (i.e., $(P: N)_{Cap}$ ratio of ≈ 1: 1.1).

(ii) Specific energy density measurements of ETO@nano-C//SSB@nano-C full-scale CR2032-coin LIB cells

The specific energy density (Wh/kg) of designed ETO@nano-C//SSB@nano-C full-scale LIB cells can be calculated according the experimental sets at 0.1C, and 25 ^{o}C, according the following relationship:

- The specific energy density (Wh/kg) of full-cell ETO@nano-C//SSB@nano-C LIB at 0.1C = the average operation voltage (V) x discharge capacity value of full cell (Ah/kg) = 1.98V x 168.68 (Ah/kg) = 333.99 (~324) Wh/kg.
- However, the specific energy density for the full-cell 3D-LFPO@nano-C-mutated-LIB at 0.1C= 1.98V x 168.68(Ah/kg) x 0.496= 165.66 Wh/kg.

Where the average operation voltage (V) is 1.98 V, the discharge capacity value is 168.68 mAh/g, and the mass fraction of cathode (P-electrode), as an individual component in one SSB@nano-C, is approximately 49.6% (see Figure S2)

S3. Large scale, collar packing of CR2032-coin cell sets in pouch LIB-model

(i) The mass fraction analysis of designated pouch LIB models

The pouch LIB models, the stacking layers of super-open-mesoeye 3D-LFPO@nano-C (such as SSB@nano-C, MS@nano-C and DCS@nano-C) cathode P-electrodes, and ETO@nano-C anode N-electrode are controlled according the depicted contents used for formation the model. Figure S2 shows the mass composition ratio of cathodic materials used for working P-electrode fabrication is 75% from the total content. However, the content ratio used for fabrication of P-electrode is 0.75: 0.15: 0.10, for cathodic materials (SSB@nano-C): C: PVDF linker, respectively. The SSB@nano-C cathode P-electrodes, and ETO@nano-C N-electrodes connected and arranged in the CR2032 coin cells and then packed into a collar-like shape (Scheme 1). The mass fraction of cathode as an individual component in pouch cell is approximately 49.6%, where the total LIB cell mass equals 6 g (0.006 kg) see Figs. S2.

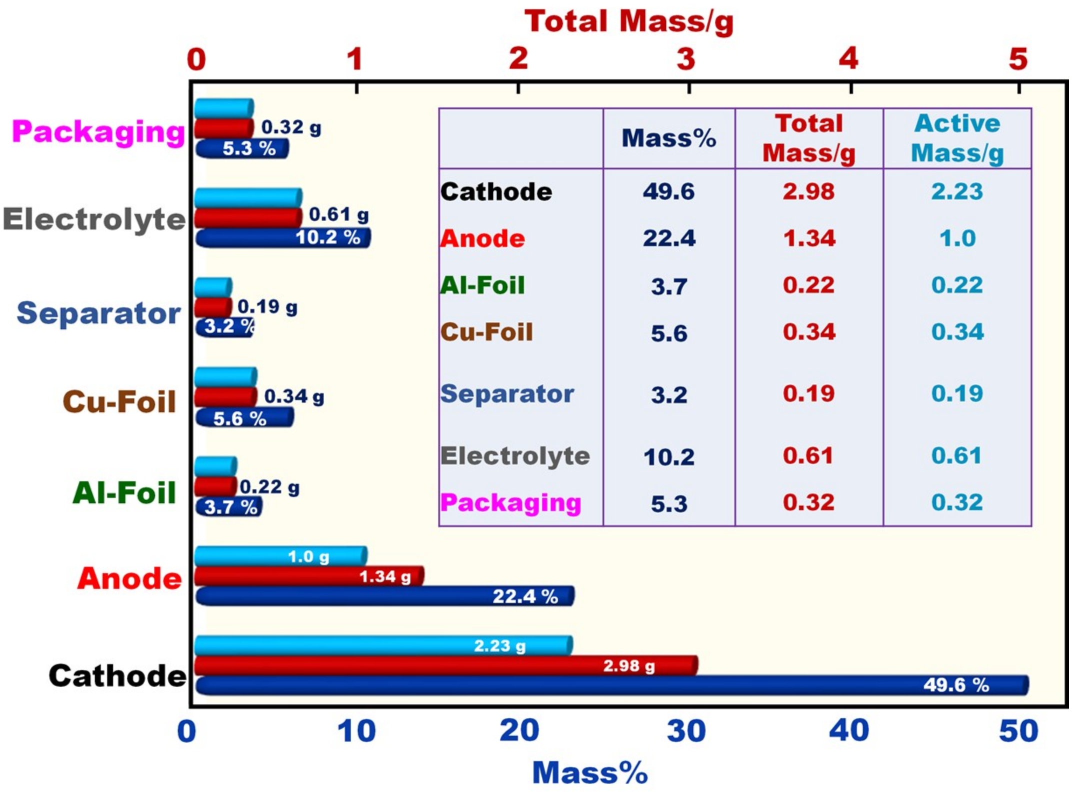

Fig. S2 Schematic diagram of the pouch cell used for the weight fraction calculation, and the mass fraction of individual components used in the formation of ETO@nano-C//SSB@nano-C anode//cathode pouch LIB-model.

(ii) Electrochemical parameters of cell battery pouch LIB-models

The ETO@nano-C (N-electrode)//SSB@nano-C (P-electrode) stacked in pouch LIB-model is designed with specific 3D dimensions of 35 mm (width), 55 mm (length) and ~2.5-3mm (thickness), respectively. The stacked-layers of ETO@nano-C anode (N) // SSB@nano-C-cathode (P) electrodes are designed in pouch LIB-models by control the $(N/P)_{Cap}$ balancing ratios with 5-/6-layers with 10 sides for Cu- electrode and Al-electrode, respectively. Well-packed and dense ETO@nano-C anode//SSB@nano-C-cathode coin cells are contiguously connected into a series of the stacking layer configurations of pouch LIB-types. As shown in Figure S2, we consider the mass component and fraction constitutes to control the pouch LIB-design. Accordingly, the actively-loaded mass in the stacking-layer SSB@nano-C (cathode) is 2.23 g, and ETO@nano-C (anode) is 1.0 g in pouch LIB-types.

To optimize the pouch LIB-design, the electrode area is selected with the following dimensions of ($3*5=15\ cm^2$) and ($3*4.75 = 14.3\ cm^2$) for SSB@nano-C cathode and ETO@nano-C anode, respectively. Thus, the total area of the SSB@nano-C cathode and ETO@nano-C anode coverage the pouch LIB cells are 150 and 143 cm^2; respectively. Therefore, the SSB@nano-C cathode and ETO@nano-C anode mass stacking is 14.87 and 6.99 mg/cm^2, respectively. Accordingly, the areal discharge capacity the SSB@nano-C cathode and ETO@nano-C anode is 2.51 and 1.18 Ah/cm^2, respectively.

To evaluate the pouch LIB designs volumetric energy density, we used the following equation:
** Volumetric energy density = gravimetric cell energy (Wh/kg)* total cell mass (kg)/ cell active area volume (L)
Where Gravimetric cell energy = 165.66 Wh/kg; total cell mass = 6 g = 0.006 kg; active area volume = 35 mm (width)* 55 mm (length)* 2.4 mm cell thickness = 4620 mm^3 = 0.00462 L
Thus, the volumetric energy density =165.66 * 0.006 / 0.00462 = 215.14 Wh/L

S4. Mesoscopic super-open-eye MS@nano-C and DCS@nano-C cathode materials

Mesoscopic super-open-eye MS@nano-C and DCS@nano-C cathode materials are investigated using FE-SEM, EDS, and HR-TEM analysis as shown in Figs S3 and S4; respectively. The crystal structures of MS@nano-C and DCS@nano-C super-open-eye MS@n5.3ano-C and DCS@nano-C cathodic structures are further analyzed by HR-TEM as shown in Figs. (S3 (d-f) and S4 (e-g)); respectively. MS@nano-C sample exhibits crystal planes with interatomic spacing of (0.426, 0.38) nm, assigned to {-101} and {210} planes; respectively, Fig.S3 (e). The DCS@nano-C-structure shows crystal planes with interatomic spacing of (0.458, 1.05) nm, assigned to {001} and {100} planes of orthorhombic olivine structure; respectively, Fig. S4 (f). SAED (selected area electron diffraction) patterns of MS@nano-C and DCS@nano-C cathode particles can be shown in Figs. (S3 (f) and S4(g)); respectively. It is evident that, exposed [-12-1] and [010] planes are prominent for prepared heterogeneous MS@nano-C and DCS@nano-C spheroid cathode particles; respectively. HR-TEM photos provide clear thin and smoothed C-shell layer (2-4 nm) covering the surface Figs. (S3(d-inset) and S4(f)), which are also characterized by Raman spectra. The elemental mapping was performed based on the Energy-dispersive X-ray spectroscopy (EDX) measurement to investigate composition distribution of the composite chemical contents along the super-open-eye MS@nano-C and DCS@nano-C cathode materials by using a 200 kV TEM (JEOL 2100F, JEOL Ltd) field emission-type transmission electron gun microscope as shown in Fig. S3(c) and S4(d). HR-TEM and STEM-EDS results indicate the purity of MS@nano-C and DCS@nano-C samples as pure LiFePO4 cathode composites with no present of other foreign elements.

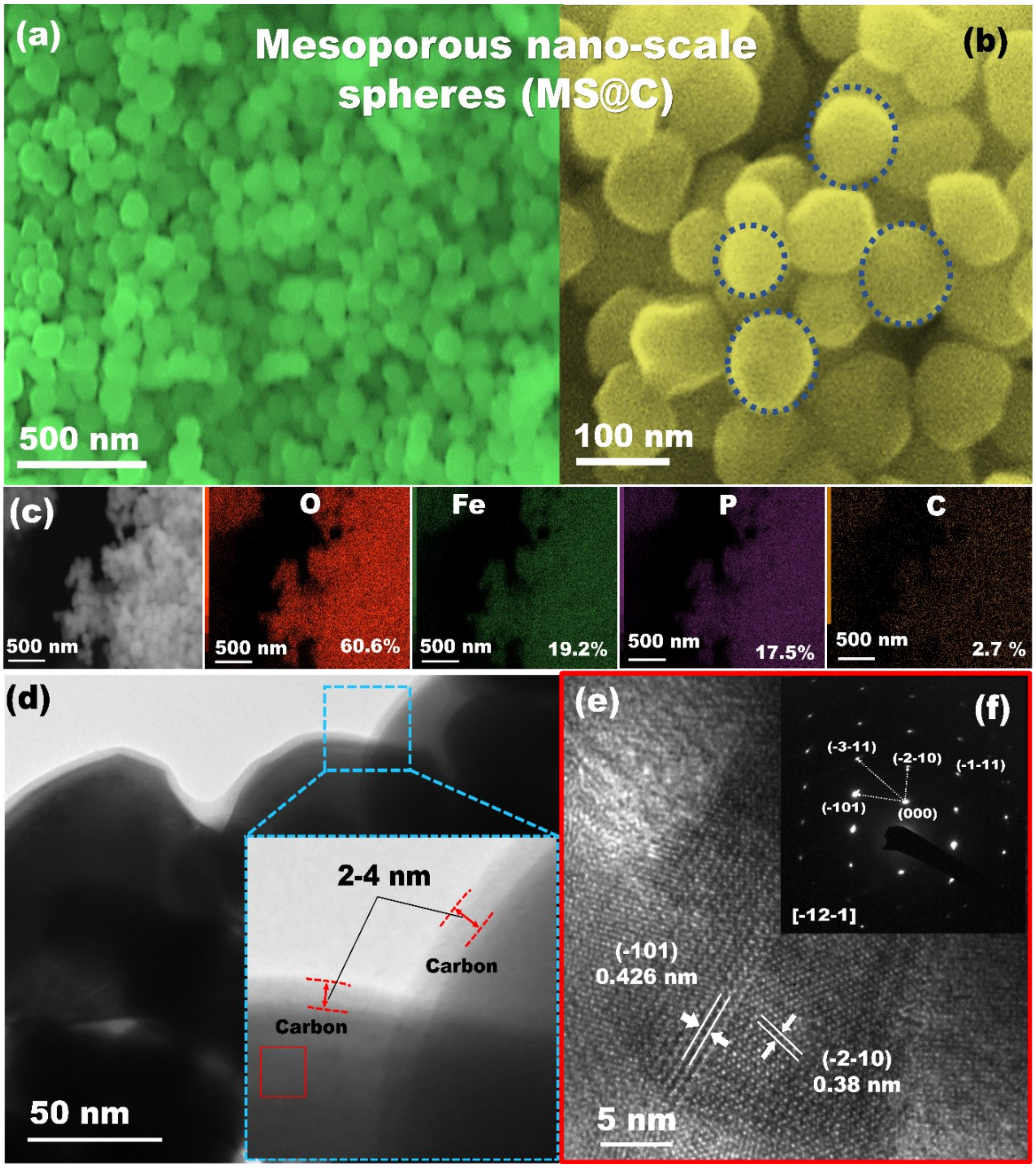

Fig. S3 Microscopic analysis patterns based on FE-SEM (a-b), EDX (c), HR-TEM (d-e), and ED (f) images of mesoscopic supper-open-eye core/shell spheroid MS@nano-C cathode. (f) The selected area of ED pattern image recorded along the [-12-1] plane of MS@nano-C geometrics.

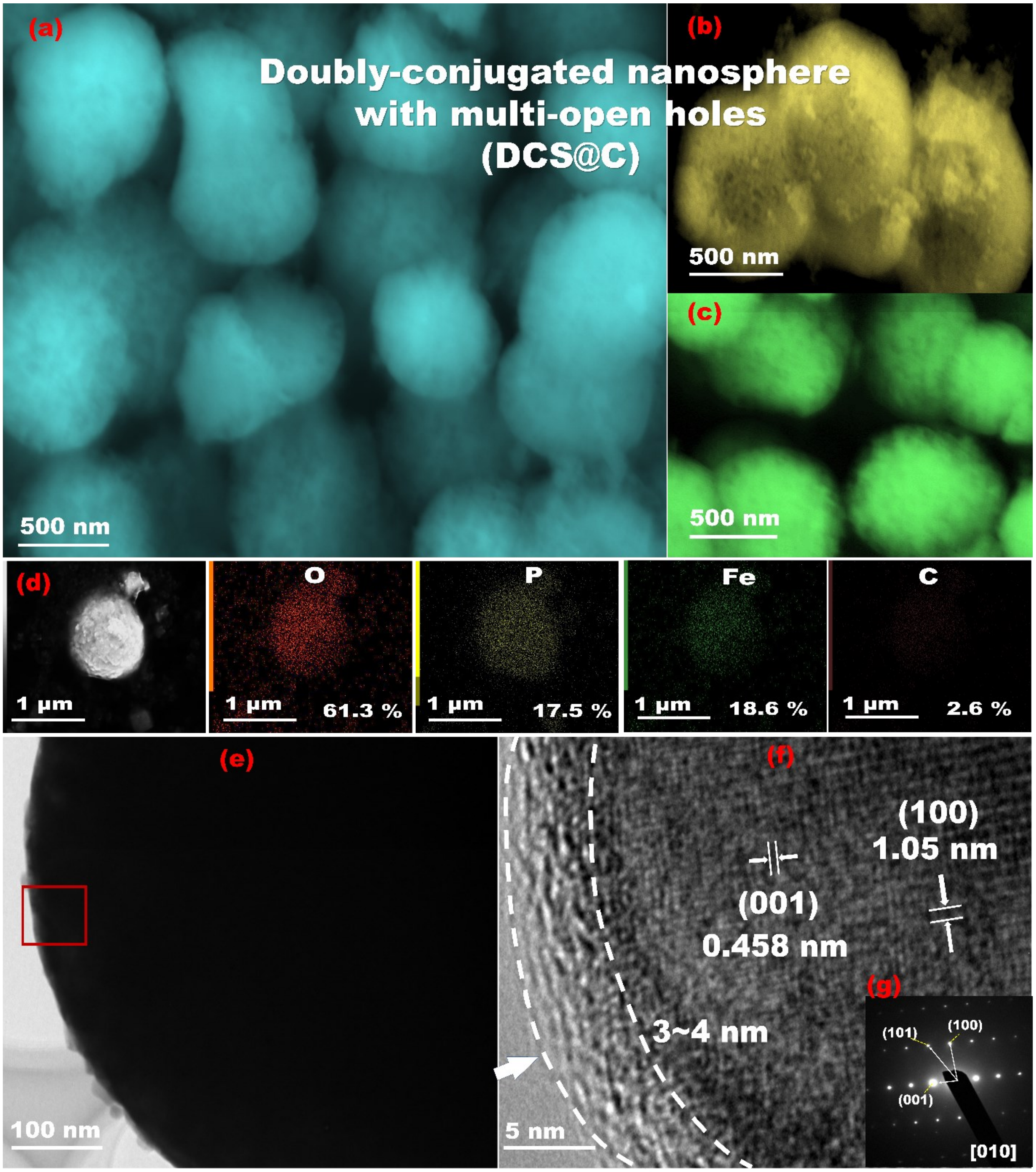

Fig. S4 Microscopic analysis patterns based on FE-SEM (a-b), EDX (c), HR-TEM (d-e), and ED (f) images of mesoscopic supper-open-eye core/shell spheroid DCS@nano-C cathode. (f) The selected area of ED pattern image recorded along the [010] plane of DCS@nano-C geometrics.

S5. Textural parameters of mesoscopic supper-open-eye core/shell spheroids

We measured the N_2 adsorption−desorption isotherms mesoscopic supper-open-eye core/shell spheroid 3DLFPO@nano-C cathodes to determine the specific surface areas (S_{BET} m^2/g) of mesoporous spheroid structures, and pore size using non-linear density functional theory (NLDFT), SI-Fig.S5(a, b). Our finding indicated that the N_2 isotherms featured a type IV with H_2 hysteresis loop for all tested cathode samples. This finding indicates the formation of mesocage caves and high surface coverages of the entire spheroid-structures. The spheroid design, active facet-surface type orientation, variable model structures, and nano -particles dressed along the particles surface are affected the textural parameters, dynamic arrangement of space holes, mesocages and pore entrances, and surface coverage area. For instance, the (S_{BET}, m^2/g) value decreases in this order SSB@nano-C > MS@nano-C > DCS@nano-C spheres, respectively. Among all prepared spheroid structures, the SSB@nano-C design can be considered as diverse surface texture electrode in terms of large surface–to-pore volume ratios, uniformly-arranged cage cavity and well-ordered entrances for long-timescale stability, excellent specific capacities, high-energy- density, and high LIBs electrochemical performances. The calculated surface areas are 294.5, 81.0 and 13.1 $m^2.g^{-1}$ and the corresponding pore size diameters are 12.52,44.69; 13.98,- and 16.67,52.74 nm for @nano-C, @nano-C and @nano-C, respectively.

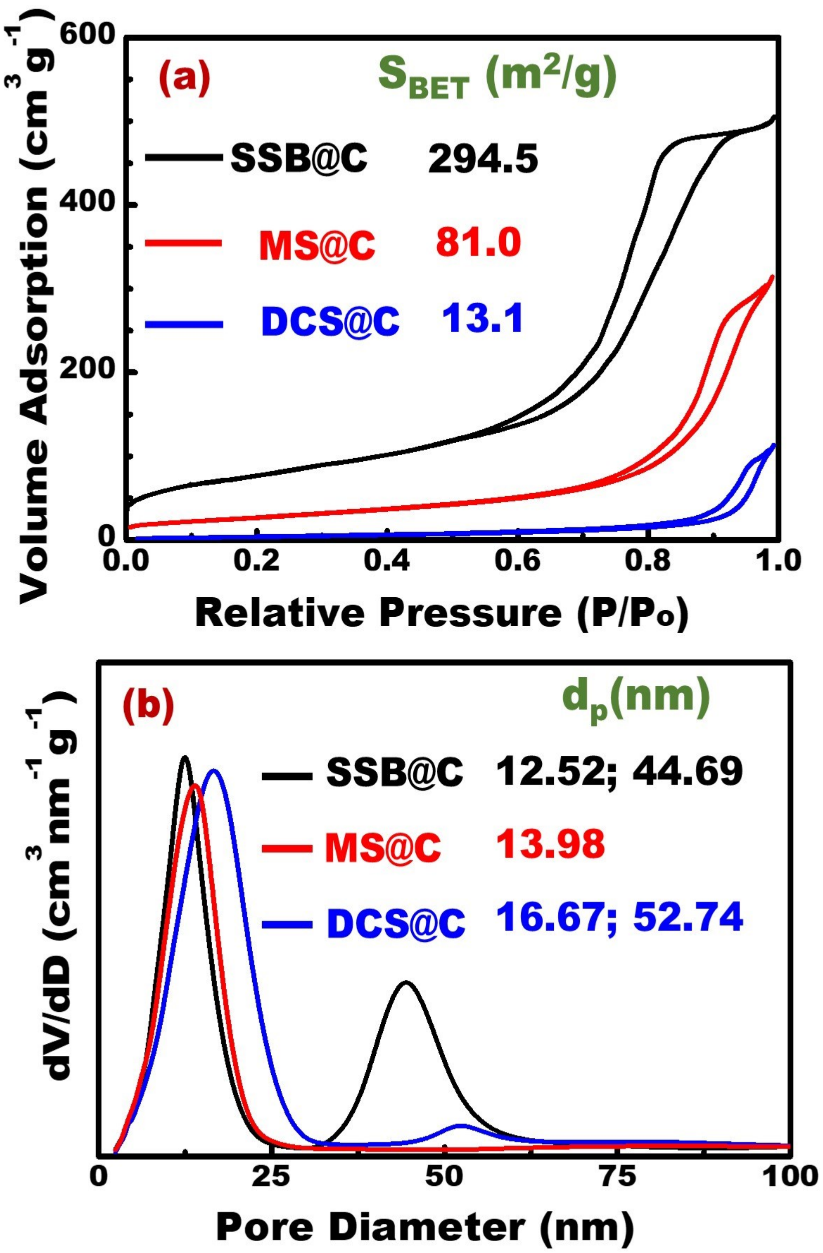

Fig.S5 **(a)** Nitrogen adsorption-desorption isotherms, including summary of surface areas measurements, and **(b)** pore-size features of SSB@nano-C, MS@nano-C and DCS@nano-C cathode-materials.

S6. Thermal stability of mesoscopic supper-open-eye core/shell spheroids

Thermal stability of mesoscopic supper-open-eye core/shell spheroid cathodes is shown in Fig. S6. The curves of TGA analysis indicated that all testes mesoscopic supper-open-eye core/shell spheroids have the same trends with slight changes in the weight loss based on the material structure. The TGA profiles show the first region below 300°C that can be attributed to the vaporization of absorbed H_2O molecules. The second region from 300°C to 520°C associated with the oxidation of carbon coated samples mainly due to pyrolysis of the organic compounds. (iii) The third region above 520°C to 900°C slightly changes in the weight loss, indicating the impurity phase disappeared and structure conversions. The DSC analysis of SSB@nano-C displays a clear endothermic peak around 435°C that reflects a maximum rate of reaction. In the range above 600°C there is not any peak discovered indicating the termination of the oxidization reactions of SSB@nano-C material.

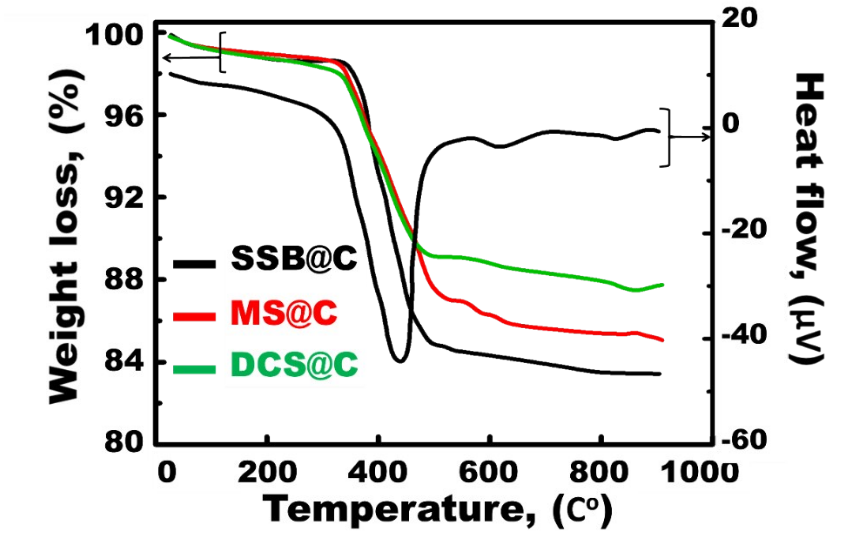

Fig.S6 TG curves of the different mesoscopic supper-open-eye core/shell spheroids of SSB@nano-C, MS@nano-C, and DCS@nano-C cathode composites, and the DSC@nano-C analysis of SSB@nano-C.

S7. Chemical structure of mesoscopic supper-open-eye core/shell spheroid cathodes

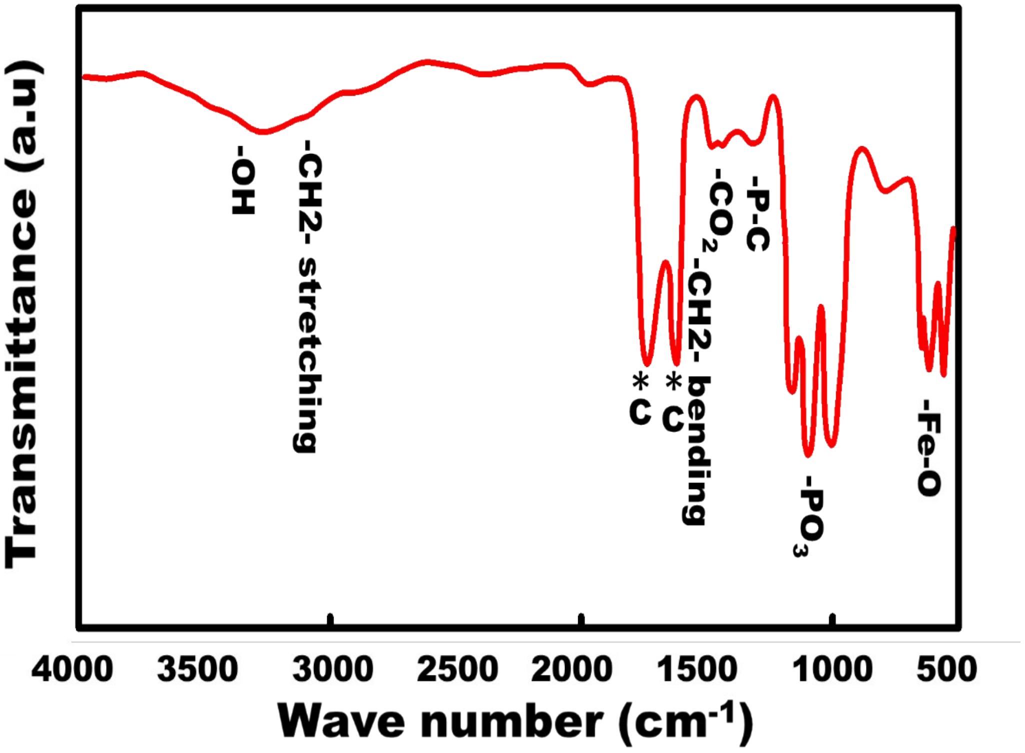

Fig. S7 FT-IR spectra of mesoscopic supper-open-eye core/shell spheroid cathodes.

To investigate the material functionality of mesoscopic supper-open-eye core/shell spheroid cathodes, Fig. (S7). The FTIR spectrum indicated the presence of multi peaks of the tested SSB@nano-C cathode material. The peaks at 578 cm^{-1}, small intense peak around 1440 cm^{-1} and peak at 3250 cm^{-1} that indicated to Fe–O, P–C and stretching vibration of $-CH_2-$ group. The PO_4^{3-} anions has multi-peaks associated with its vibration mode, symmetric stretching mode ν_1 appear at 972 cm^{-1}, the ν_2 mode around 510 cm^{-1}; and the ν_3, ν_4 modes in the region 1051-1093 cm^{-1}. The area around 3250 cm^{-1} is sensitive to Li localization region of Li_3PO_4. The weak peak at 3350 cm^{-1}, 1536 cm^{-1} , and 1615 and 1718 cm^{-1} can be attributed to the existence of–OH group, CH_2-bending vibration, and C=O stretching, respectively.

S8. Chemical bonding and composition of mesoscopic supper-open-eye core/shell spheroid cathodes

Raman spectroscopy enables investigation of surface composition of mesoscopic supper-open-eye core/shell spheroid anode/cathode. In this regards, Raman spectroscopy of SSB@nano-C composite material is recorded with 10 scans of 5s in the range of 500-2000 cm^{-1}, as shown in **Fig.S8.** The bands at 631.0 and 948 cm^{-1} agree to symmetric mode of Fe_3O_4 and PO_4^{3-}groups in SSB@nano-C. Two peak mounts at 1346 and 1602 cm^{-1} are attributed to D and G bands of nano-C in SSB@nano-C, respectively. The C-D-peak match to a disordered carbon of highly defective graphite and the C-G- peak is related to (graphite, in-plane vibrations with E_{2g} symmetry). Therefore, according to Raman and FT-IR analysis, it confirms the present of thin carbon layer on surface of SSB@nano-C which is partially cross-linked via C=C, C=N, C=O bonds after carbonization.

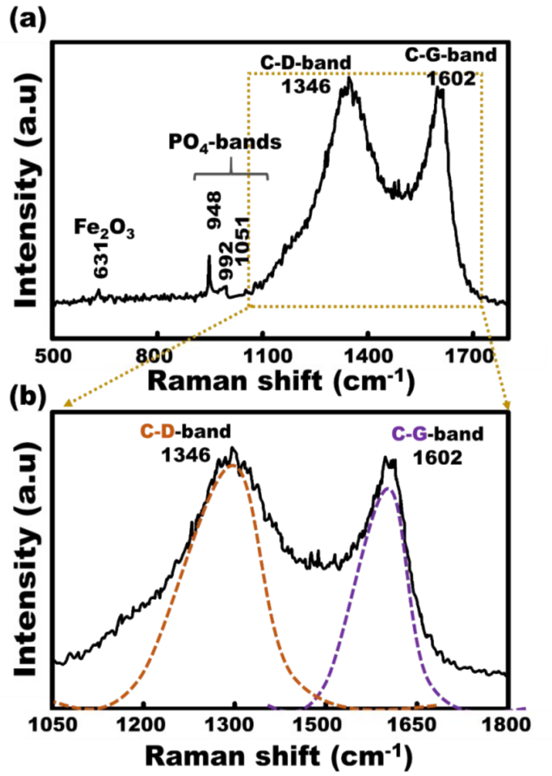

Fig. S8. (a and **b)** Raman spectra of mesoscopic supper-open-eye core/shell spheroid SSB@nano-C cathodes.

S9. Surface analysis of super-open-mesoeye LFPO@nano-C cathode materials

XPS analysis is investigated the materials compositions, oxidation states and valences of 3D-LFPO@nano-C cathode materials such as SSB@nano-C geometrics (Fig. S9). The XPS pattern of super-open-mesoeye LFPO@nano-C materials shows different peaks at 711.3 and 530.9 eV. These peaks are the BEs related to Fe2p and O1s, respectively, where Fe-BE related only to its valency state (Fe^{2+}). Some other valence states couldn't observe. P2p, Li1s and C1s peaks are detected at 133.3, eV 54.5 and 285.2 eV, respectively, indicating the chemical state of LFPO cathode. The XPS results of olivine $LiFePO_4$@nano-C composition are in agreement with XRD profiles.

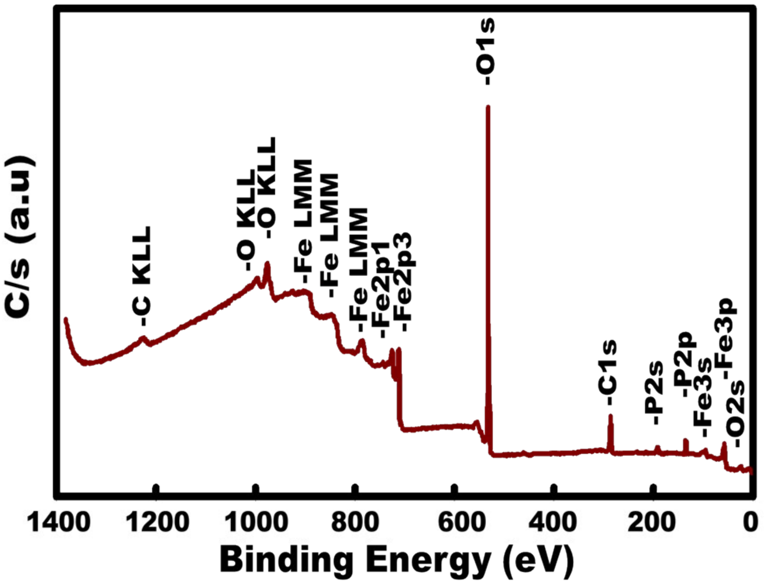

Fig. S9 XPS survey spectrum of super-open-mesoeye LFPO@nano-C cathode composites.

S10. Crystal Structure formation of super-open-mesoeye anode/cathode

The crystal structures of super-open-mesoeye SSB@nano-C, MS@nano-C and DCS@nano-C (cathodes) and ETO@nano-C (anode) materials are investigated by using XRD analysis (Fig. S10). The XRD diffraction peaks of uper-open-mesoeye SSB@nano-C, MS@nano-C and DCS@nano-C (cathodes) can be readily indexed to the well-defined pure orthorhombic olivine-LFPO geometrics with Pnma symmetry and lattice units of a = 9.814 Å, b = 5.817 Å, and c = 4.783 Å (in addition to the unit volume of 286.95$Å^3$). These lattice unit parameters are matched with JCPDS card No. 83-2092 [Ref.S1, Ref.S2]. The super-open-mesoeye 3D-LFPO geometrics with [010] plane indicates the feasible Li^+-ion lithiation and delithiation pathways for superb electron-ion kinetics and fast ionic diffusion during discharge-charge cycle process, see Figure S10 (b). The SSB@nano-C crystal structure shows highly-exposed *ac*-plane orientation, leading to formulate low surface energy topologies for fast electrons/ Li^+-ions diffusion dynamics within lithiation/delithiation process. Figure S10c shows diffraction peaks of ETO@nano-C anode with tetragonal structure with $I4_1$/amd space symmetry. The XRD pattern indicates the formation of TiO_2-anatase structure with lattice units (a= 3.789 Å, b= 3.789 Å and c = 9.489 Å), corresponding to JCPDS 21-1272. These findings provide evidence of the structurally-stable anode/cathode electrodes with unique atomic-scale organizations.

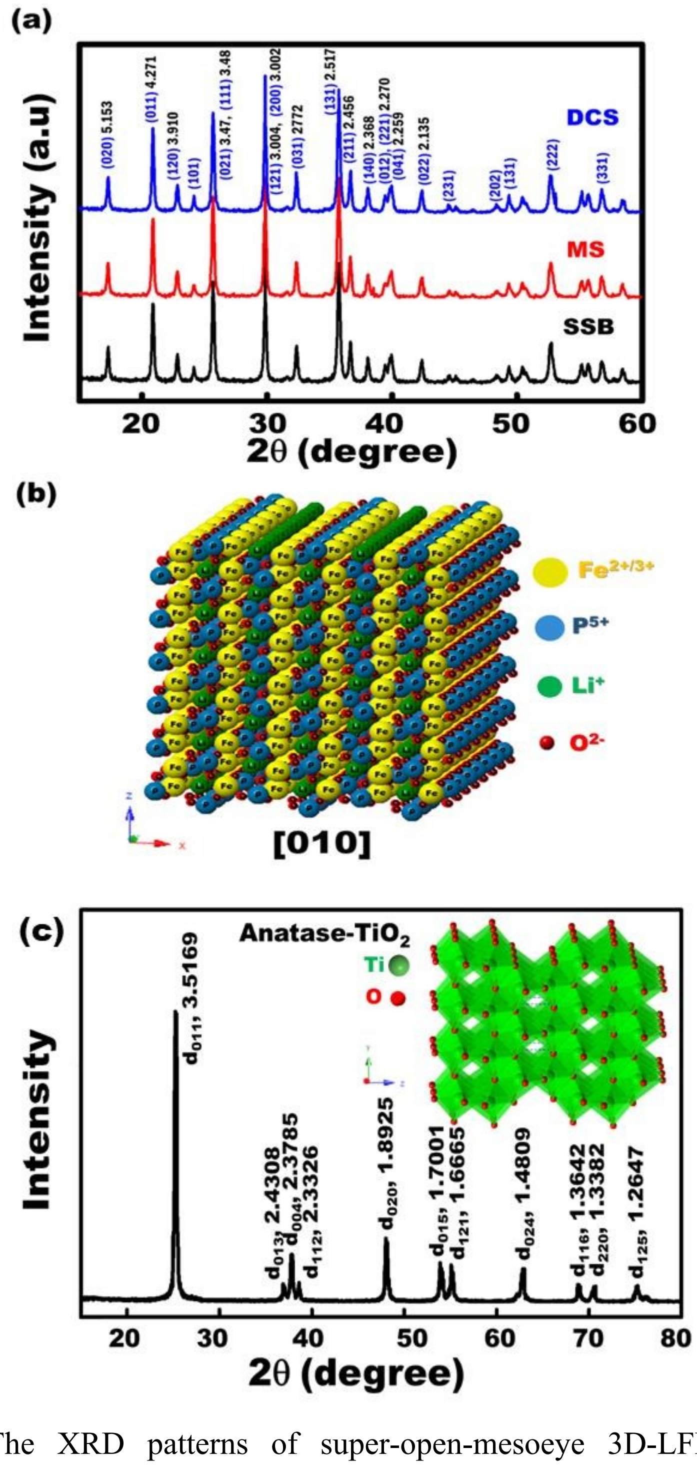

Figure S10 (a) The XRD patterns of super-open-mesoeye 3D-LFPO@nano-C such as SSB@nano-C, MS@-nano-C, and DCS@nano-C cathode materials. (b) 3D-LFPO structures with [010] plane and its feasible Li^{+}-ion lithiation and delithiation pathways. (c) The XRD patterns of the open-mesoeye ETO@nano-C anatase structure (c).

S11. Stability of anode and cathode structures for fully-reversible cycles

We investigate the stability of anode and cathode structures for fully-reversible cycles through the microscopic analyses using SEM and EDX profiles of anode and cathode material structures after multiple 100th cycles (Figure S11).

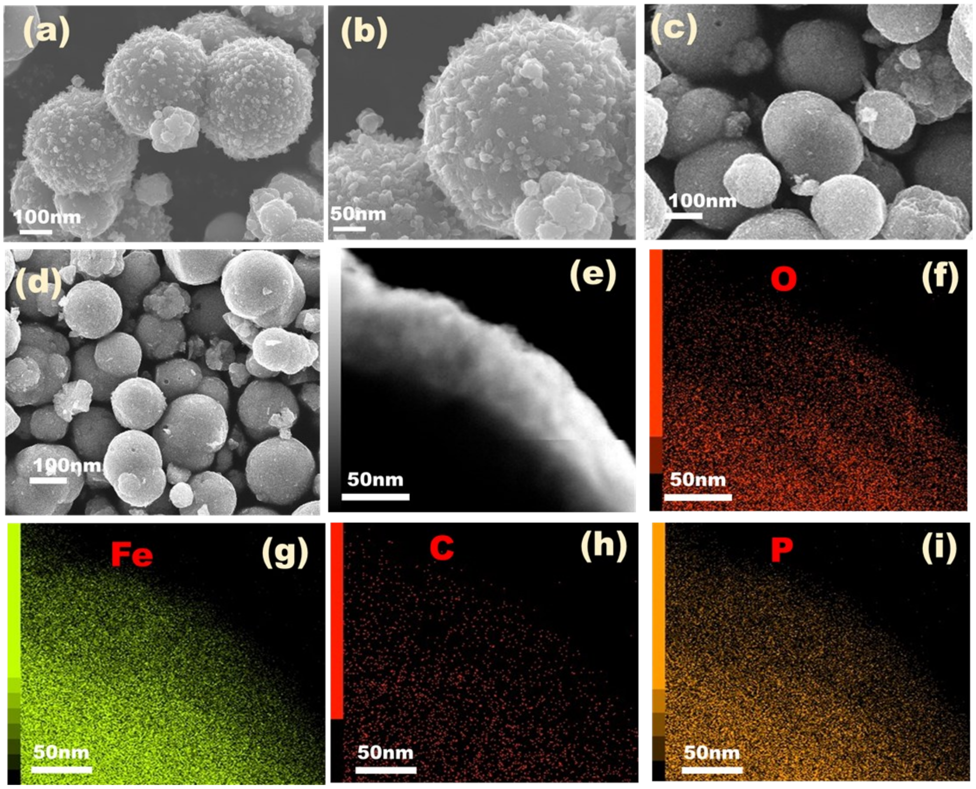

Figure S11 SEM and EDX patterns of SSB@nano-C cathode material structures after multiple 100th cycles.

Overall, the electrochemical cycling performance analysis clarified the stability of structurally-stable anode/cathode electrodes designed in half- or full-cell cathode LIB-CR2032 designs. The retention of anode/cathode structure geometry and surface topology leads to full dynamic LIB storage and accommodation systems, in which heavily loaded Li^+-ion diffusion, and minimized electron transport distance are characterized for cycled electrodes. The electrode uniqueness in its sustainability of electronic conductivity would enhance the rate capability, and kinetic Li^+-ion transport efficiency during the lithiation (discharge)/delithiation (charge) processes. For instance, the microscopic patterns show the stability of SSB@nano-C material designed in half-and full-cell LIB-CR2032 designs. This finding leads to stable design configurations due to the retention of functional surface interfaces of spheroids/cuboids and its rich spatial distribution in complexity,

anisotropy, and heterogeneity. The patterns of cycled cathode materials show the retention of mesoscopic supper-open-eye core/shell spheroids, and interiorly uniform accommodation/storage space pockets (i.e., surface mesogrooves and mesoeye entrances, interior innumerable caves and core hollowness-like nests), and the 6-facet cuboid-capped gradients that indicate the functional ability of the SBB@nano-C module in simultaneous, full-scale LIB models. Furthermore, the stability of well-and large-scale surface dispersion of thin C-shell dressings along SBB@nano-C cathode layers plays an important role in preventing Fe dissolution or atomic dislocation (i.e., well-structured robustness) against severe treatment conditions, thereby enabling excellent sustainability of the electronic surface mobility and conductivity within cycles.

S12. Functional cell parameters of super-open-mesoeye half-, and full- cell LIB-CR2032 designs

Table S1 A comparison between the super-open-mesoeye electrodes designed in half-, and full-cell LIB-CR2032 designs and the other reported $LiFePO_4$//TiO_2 electrode functionalities.

Cathode material	Anode material	Nominal Voltage (V)	Cycles	Specific Capacity mAh/g	Coulombic efficiency	Ref
$LiFePO_4$	Anatase TiO_2 hollow nanofibers	1.4	retained 88% of its reversible capacity after 300 cycles	103	> 99 %	Ref [S3]
$LiFePO_4$	Rutile TiO_2	1.8	retained 50% of its reversible capacity after 40 cycles	150	Not mentioned	Ref [S4]
$LiFePO_4$	Anatase TiO_2	1.6	81% of its initial capacity after 300 cycles/2^0C	160	Not mentioned	Ref [S5]
$LiFePO_4$	anatase/graphe ne	1.6	700	127	~ 100%	Ref [S6]
LiFePO4	spinel $Li_4Ti_5O_{12}$/C	1.8	Retain 98.1% after 400 cycles	167	~100%	Ref [S7]
$LiFePO_4$	spinel Li_4Ti_5O12	1.65	Retain 98.9% after 100 cycles	150	~100%	Ref [S8]
SSB@nano-C	ETO@nano-C	1.98	Retains 97.2% after 2000 cycles at 1C	168.68	~100%	Current Work

Supporting References

[S1] B. Guo, H. Ruan, C. Zheng, H. Fei and M. Wei, Sci. Rep. 3(2013) p. 2788

[S2] C. Sun, S. Rajasekhara, J. B. Goodenough and F. Zhou, J. Am. Chem. Soc., 133 (2011) pp. 2132–2135.

[S3] X. Zhang, V. Aravindan, P. Suresh Kumar, H. Liu, J. Sundaramurthy, S. Ramakrishna, S. Madhavi Nanoscale, **5**, (2013) pp. 5973-5980.

[S4] J. Hassoun, M. Pfanzelt, P. Kubiak, M. Wohlfahrt-Mehrens, B. Scrosati, J. Power Sources, **217**, (2012) pp. 459-463

[S5] Z. Guo, X. Dong, D. Zhou, Y. Du, Y. Wang, Y. Xia, RSC Adv., **3** (2013) pp. 3352-3358.

[S6] D. Choi, et al., Electrochem. Commun. **12(3)** (2010) pp. 378-381.

[S7] C-C.Yang, H-C. Hu, S.J. Lin, W-C. Chien, J. Power Sources, **258**, (2014) pp. 424-433.

[S8] J. Morales, R. Trocoli, S. Franger, J. Santos-Pena, Electrochim. Acta 55 (2010) pp. 3075e3082.
